# Inflammatory Bowel Disease: Mechanisms and Therapeutic Advances

**DOI:** 10.1002/mco2.70914

**Published:** 2026-08-16

**Authors:** Andrea Papait, Anna Cargnoni, Franco Scaldaferri, Loris Lopetuso, Antonietta Rosa Silini, Ornella Parolini

**Affiliations:** ^1^ Dipartimento di Scienze della Vita e Sanità Pubblica Università Cattolica del Sacro Cuore, Largo Francesco Vito 1 Rome Italy; ^2^ Fondazione Policlinico Universitario A. Gemelli IRCCS Rome Italy; ^3^ Centro di Ricerche Eugenia Menni, Fondazione Poliambulanza Istituto Ospedaliero Brescia Italy; ^4^ UOS Malattie Infiammatorie Croniche Intestinali, Centro Malattie Apparato Digerente (CeMAD), Dipartimento di Scienze Mediche e Chirurgiche, Fondazione Policlinico Universitario "A. Gemelli" IRCCS, L.go A. Gemelli 8 Rome Italy; ^5^ Dipartimento di Medicina e Chirurgia Traslazionale Università Cattolica del Sacro Cuore, L.go F. Vito 1 Rome Italy; ^6^ Fondazione IRCCS Casa Sollievo della Sofferenza San Giovanni Rotondo, Foggia Italy

**Keywords:** fecal microbiota transplantation, inflammatory bowel disease (IBD), innate and adaptive immunity, mesenchymal stem/stromal cells (MSCs), microbiota alterations, precision medicine

## Abstract

Inflammatory bowel disease (IBD), which includes ulcerative colitis and Crohn's disease, represents a chronic, immune‐mediated pathology in which genetic susceptibility, environmental exposures, and microbial perturbations converge to destabilize intestinal homeostasis. Despite a steady global rise in incidence affecting more than 10 million people worldwide, current single‐pathway biologic and small‐molecule therapies still leave 30–40% of patients without durable remission, with progression to stricturing, fistulizing, or transmural complications. Yet the mechanistic integration of innate and adaptive immune networks, epithelial barrier dysfunction, and microbiota‑driven inflammation remains fragmented. This review summarizes recent advances in understanding the pathophysiological mechanisms driving IBD, focusing on innate and adaptive immune networks. Current therapies, including cytokine or integrin‐targeting biologics and small‐molecule inhibitors like Janus Kinase (JAK) antagonists, are critically evaluated in light of their restricted pathway coverage. In response to these challenges, emerging strategies are examined targeting nonimmune pathways, including microRNA‑124‐mediated gene regulation, fecal microbiota transplantation, and mesenchymal stromal cell therapies that combine immunomodulation with tissue repair. The development of effective IBD therapies is likely to benefit from combining complementary treatment strategies, guided by molecular and cellular profiling. This review underscores a paradigm shift from monotherapies to integrated, multitarget approaches as the new frontier in IBD management.

## Graphical abstract

Inflammatory bowel disease (IBD), encompassing ulcerative colitis and Crohn's disease, is a rising, multifactorial chronic disorder driven by dysregulated mucosal immunity in genetically susceptible individuals and influenced by environmental and microbial factors. Despite advances in targeted therapies, disease incidence, especially pediatric onset, is increasing worldwide and clinical outcomes remain suboptimal, underscoring the need for mechanism‐based, durable treatments and improved patient stratification integrating immunome, genome, exposome, and microbiome insights

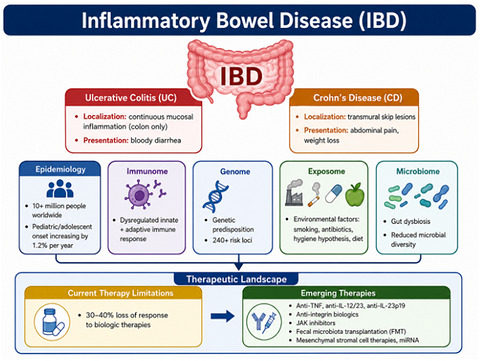

## 1 Introduction

Inflammatory bowel disease (IBD) is a chronic immune‐mediated disorder affecting the gastrointestinal tract, presenting as ulcerative colitis (UC) or Crohn's disease (CD), each distinct in clinical presentation, disease localization, and molecular pathology [1, 2, 3].

Over the past five decades, IBD incidence and prevalence have risen in both developed (Europe and North America) and developing (Asia and Latin America) regions, with recent epidemiological data showing significant increases among children and adolescents [4].

More than 10 million people worldwide are estimated to live with IBD as of 2024, with age‐standardized prevalence exceeding 0.3% in most high‐income countries and rising rapidly in newly industrialized regions of Asia, South America, and Africa [5] (GBD 2021 IBD Collaborators 2024). Pediatric‐onset disease is the fastest‐growing demographic, expanding by approximately 1.2% per year, with implications for lifetime disease burden and healthcare‐system planning. Despite substantial therapeutic progress, real‐world outcomes remain suboptimal: 30–40% of patients with CD still require intestinal resection within 10 years of diagnosis, and 30–40% of patients on biologic therapy lose response within 1–3 years [6]. These figures underscore the urgent unmet need for mechanism‐based therapies that achieve durable, deep remission across the heterogeneous IBD population.

Although the precise etiology remains elusive, IBD is now recognized as a multifactorial disease primarily driven by dysregulation of the intestinal immune system in genetically susceptible individuals and triggered by environmental and microbial factors. Disruption of the gut microbiota is frequently observed in IBD patients, although the causality of this dysbiosis remains under investigation. Genome‐wide association studies have identified over 240 IBD risk loci concentrated on immune‐regulatory genes, epithelial barrier components, and microbial‐sensing pathways [7], reinforcing the view that IBD arises from an aberrant mucosal immune response to the gut microbiota in genetically predisposed individuals. Parallel advances in microbiome research have consistently documented dysbiosis in IBD patients, with altered composition and reduced diversity of gut microbial communities, although the causal role of specific taxa and functional shifts is still under active investigation.

Epidemiological evidence supports the hygiene hypothesis, linking the rise in autoimmune and chronic inflammatory disorders [8], among which IBD, to factors like decreased infectious diseases, lack of parasites, antibiotic use, vaccinations, and improved sanitary conditions. In addition, some clinical observations suggest a connection between an unbalance of gut microbiota and a predisposition to immune‐mediated diseases. For example a mechanistic link has been found between childhood asthma and Helicobacter pylori colonization [9], a condition that can be aligned with the pathogenesis of CD and UC [10].

It is now clear that the pathogenesis of IBD is multifactorial, involving a complex and poorly understood interplay between genetic predisposition, environmental risk factors (smoking, antibiotic use, and diet), immune dysregulation, and alterations in the gut microbiota. It is believed that IBD arises from an abnormal immune response toward the intestinal microbiota in genetically predisposed subjects.

This multifactorial interplay explains the variability in disease course and clinical outcomes, which often diverge independently of IBD subtype [11, 12, 13, 14]. IBD is characterized by a heterogeneous clinical presentation that includes episodes of abdominal pain, chronic diarrhea, rectal bleeding, and weight loss, often associated with systemic symptoms such as fatigue and occasionally fever. Some patients experience a benign course, others face relapses, while some present with an aggressive phenotype marked by complications such as strictures, fistulas, perianal disease, dysplasia, malignancy, and/or extra‐intestinal manifestations [15], resulting in substantial healthcare costs and placing a strain on healthcare systems. This heterogeneity complicates prognosis and management and highlights the need for improved disease stratification and personalized therapeutic strategies.

**FIGURE 1:**
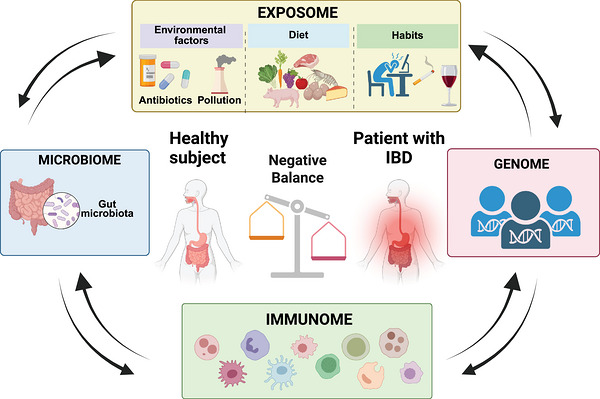
Central hubs in the complex pathogenesis of inflammatory bowel disease. Inflammatory bowel disease (IBD), which includes Crohn's disease and ulcerative colitis, is a chronic immune‐mediated disorder driven by aberrant immune responses. The pathogenesis of IBD is governed by a complex network of genes (genome), environmental factors (exposome), microbes (microbiome), and immune cells (immunome), orchestrated by central hubs that may consist of genes, proteins, metabolites, microbial products, signaling molecules, or enzymes. This intricate network underlies the diverse clinical manifestations and complications of IBD, including strictures, fistulas, and malignancies.

The intricate pathogenesis of IBD and its clinical heterogeneity likely contribute to the limited efficacy of current treatment strategies. Existing therapies range from broad‐spectrum immunomodulators, such as aminosalicylates and glucocorticoids, to biologic agents that target specific immune cells or single inflammatory pathways central to chronic inflammation, a defining feature of IBD. These biologic agents work by neutralizing inflammatory cytokines (e.g., anti‐Tumor Necrosis Factor (TNF) agents), inhibiting proinflammatory signaling cascades (e.g., Janus kinase [JAK] inhibitors), or regulating immune cell migration. While effective for many patients, a significant subset either fails to respond or loses therapeutic benefit over time, highlighting the need for more comprehensive approaches. It is now evident that IBD cannot be cured without taking into account the intricate interplay among systemic and mucosal immune systems (immunome) [16], together with the other components contributing to IBD pathogenesis: exposome (external and internal environments) [17], genome (genetic makeup) [18], and microbiome (gut‐dwelling bacteria, fungi, viruses, and archaea) [19] (Figure 1).

This review aims to summarize the current understanding of IBD pathogenic mechanisms, with particular focus on the dysregulation of innate and adaptive immune networks, and to evaluate how these insights inform existing and emerging therapeutic paradigms. We dissect the immune, genetic, and microbial components of IBD pathogenesis and summarizes the pathophysiological and therapeutic distinctions between UC and CD (Section 2). We also critically examine established pharmacologic therapies (anti‐TNF, anti‐IL‐12/23, anti‐IL‐23p19, anti‐integrin biologics, and JAK/sphingosine‐1‐phosphate [S1P]‐modulator small molecules) against the modern STRIDE‐II endpoint hierarchy of clinical, endoscopic, transmural, and histologic remission and discuss emerging nonimmune strategies including microRNA (miRNA)‐124‐mediated gene regulation, fecal microbiota transplantation (FMT), and mesenchymal stromal cell (MSC) therapies (Section 3). Finally, we examine the unresolved questions of patient stratification, biologic‐failure heterogeneity, and trial‐design priorities required to translate these insights into durable, personalized IBD control (Section 4).

Through this in‐depth examination, we strive to offer valuable insights that will propel the field by introducing efficient, pioneering strategies for managing and treating IBD.

## 2 Pathogenic Mechanisms in IBD

IBD represents a complex, multisystemic disorder characterized by highly heterogeneous phenotypic expressions, arising from the dynamic interplay of immune dysregulation, genetic susceptibility, and microbiological alterations in the gut. This intricate pathogenesis underscores the need to dissect its core components: immune mechanisms, which drive aberrant inflammatory responses; genetic factors, which confer predisposition in susceptible individuals; and microbiological influences, including dysbiosis of the intestinal microbiota. Here, these three pathogenetic pillars are addressed individually to identify their roles and interactions in IBD development.

### 2.1 Dysregulation of Intestinal Immune System

Among all factors contributing to IBD pathogenesis, such as exposome, genome, and microbiome, the scientific focus has predominantly centered on understanding the immunological mechanisms driving the disease. Indeed, it is now widely accepted that an in‐depth understanding of these immunopathogenic processes is fundamental for the rational design and implementation of targeted, efficacious therapeutic strategies in IBD.

**FIGURE 2:**
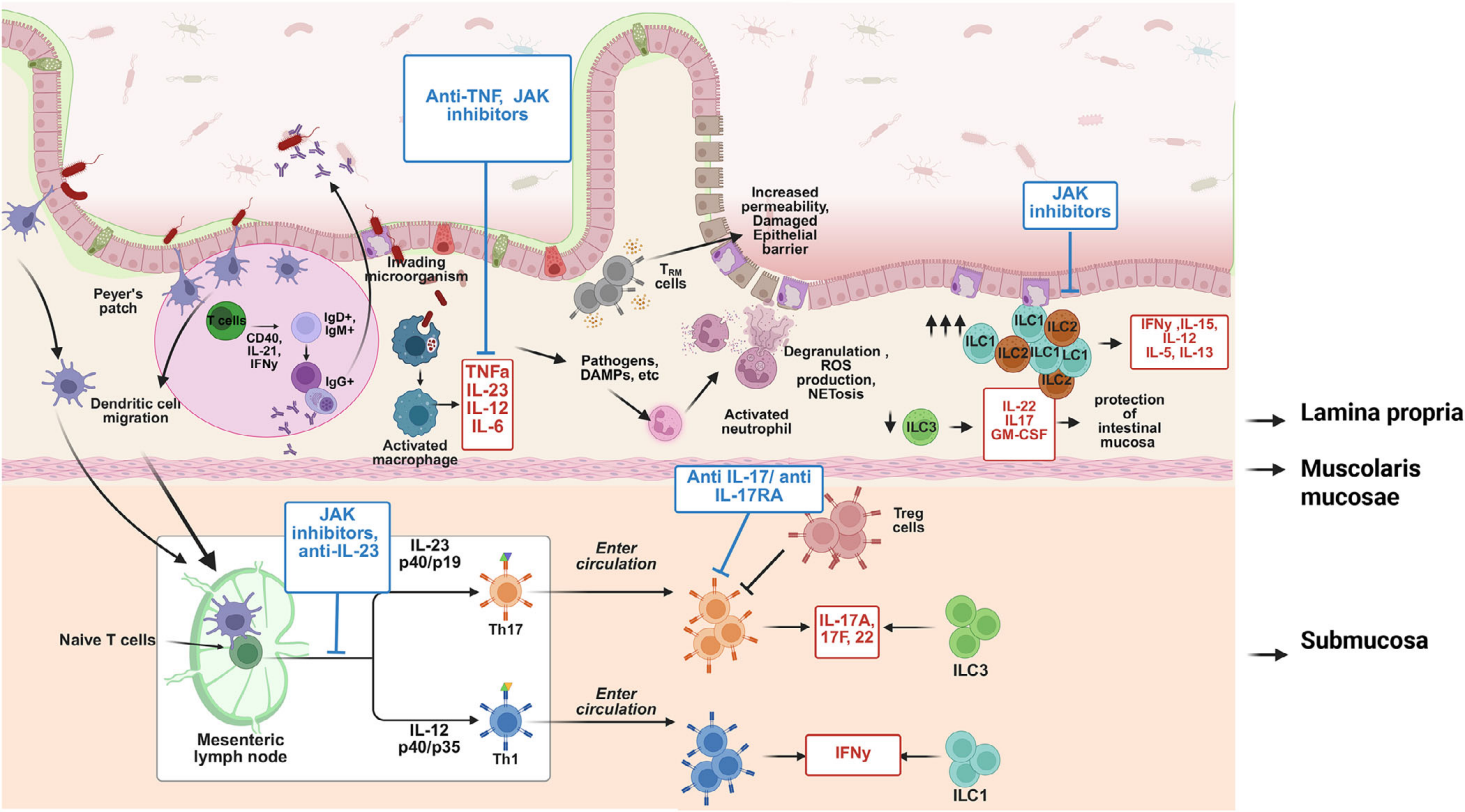
Immune dysregulation in the pathogenesis of inflammatory bowel disease. The pathogenesis of IBD, encompassing Crohn's disease and ulcerative colitis, is intricately linked to immune system dysregulation. Although genetic, environmental, and microbiome factors are involved, research predominantly emphasizes the altered immune response in IBD. Gut‐associated lymphoid tissue (GALT) plays a critical role in initiating and regulating immune responses, balancing defense against pathogens with tolerance to commensal microbiota. Dysregulation within GALT, particularly involving macrophages, dendritic cells, and B cells, leads to impaired intestinal barrier function, chronic inflammation, and tissue damage, driving the progression of IBD.

The intestinal immune system is compartmentalized both functionally (i.e. innate and adaptive arms) and anatomically, reflecting its complex role in host defense and tolerance [20]. Innate immune cells, including granulocytes and macrophages, express germline‐encoded receptors that recognize conserved microbial motifs, whereas adaptive immune cells, such as B and T lymphocytes, rely on highly variable antigen‐specific receptors. Mucosal‐associated invariant T cells bridge these systems, expressing semi‐invariant receptors [21]. Approximately 75% of the body's lymphocytes reside within the mucosal immune system, which also produces the majority of circulating immunoglobulins. These immune populations are embedded in gut‐associated lymphoid tissue (GALT), located within the subepithelial connective tissue. GALT comprises both organized structures, including Peyer's patches and isolated lymphoid follicles, and diffuse aggregates of immune cells within the lamina propria [22] (Figure 2). This system is uniquely adapted to perform dual roles: it samples luminal pathogen antigens to elicit protective adaptive responses [23], while simultaneously maintaining immune tolerance to commensal microbiota and dietary antigens [24], primarily via secretory IgA [25, 26]. This balance is essential for preserving gut homeostasis and preventing immune‐mediated pathology [27]. Disruption of GALT function has been implicated in the pathogenesis of chronic gastrointestinal diseases, including IBD, through mechanisms involving epithelial barrier breakdown and impaired mucosal repair.

Recent advances in single‐cell and spatial transcriptomics have revealed distinct immune landscapes between UC and CD. Mapping immune cell distributions in intestinal tissues could guide more precise therapies for IBD, tailored not only to disease type (CD vs UC) and lesion site (ileum, colon, rectum), but also to individual patients.

#### 2.1.1 Intestinal Innate Immune System

The intestinal innate immune system in IBD encompasses multiple cell types (e.g. neutrophils, macrophages, innate lymphoid cells [ILCs] and dendritic cells [DCs]), that contribute to inflammation and tissue dynamics [28, 29, 30]. In disease, innate immunity becomes activated when pattern recognition receptors detect pathogen‐associated molecular patterns (PAMPs) or damage‐associated molecular patterns (DAMPs) secreted from injured cells. This recognition triggers signaling not only in innate immune cells, but also in nonimmune populations such as intestinal epithelial cells and myofibroblasts, collectively amplifying immune activation and inflammation.

Neutrophils are among the first responders, rapidly migrating to inflamed sites under the influence of cytokines (e.g., IL‐1β, IL‐6, TNF‐α), chemokines (such as CCL8, CXCL10, MIP‐2), growth factors such as Granulocyte‐Macrophage Colony Stimulating Factor (GM‐CSF), Granulocyte Colony Stimulating Factor (G‐CSF) [31], and bacterial metabolites [32]. A state of neutrophil hyperactivation has been consistently observed in IBD [33, 34, 35, 36]. These cells employ a suite of antimicrobial mechanisms such as phagocytosis, degranulation, reactive oxygen species (ROS) production, and the formation of neutrophil extracellular traps (NETs), to sequester and eliminate invading pathogens. Following their effector functions, neutrophils typically undergo apoptosis and are cleared via efferocytosis.

Nevertheless, neutrophils and NETs exhibit a dual role in IBD [37, 38]. On one hand, excessive NET formation aggravates mucosal injury and inflammation, as demonstrated in animal models of dextran sulfate sodium (DSS)‐induced colitis [39]. Hyperactivated neutrophils release proteolytic enzymes (e.g., MMPs, elastase) and proinflammatory mediators (IL‐8, TNF‐α, leukotriene B4, IL‐22), which damage epithelial barrier integrity, perpetuate immune cell infiltration, and amplify inflammation through ROS‐dependent, redox‐sensitive pathways [40, 41, 42, 43, 44, 45]. Furthermore, NETs have been suggested to play a role in intestinal fibrogenesis in CD by promoting the proliferation and the collagen production of intestinal fibroblasts via the Tool Like Receptor (TLR)2/NF‐κB pathway [46]. On the other hand, neutrophils can also foster epithelial repair by releasing vascular endothelial growth factor (VEGF) and lipid mediators such as protectin D1 and resolvin E1; these factors curb further infiltration and enhance apoptotic cell clearance by macrophages [47, 48]. Accordingly, strategies aimed at modulating neutrophil lifespan and promoting their apoptosis, for example via cyclin‐dependent kinase inhibitors, represent a novel therapeutic strategy in IBD, though they are currently being studied and are not yet standard practice [49]. Meanwhile, existing biologic therapies such as the anti‐TNF monoclonal antibody infliximab and the anti‐integrin agent vedolizumab mitigate inflammation indirectly by reducing neutrophil recruitment to the intestinal mucosa [50, 51]. A more direct approach is provided by the leukocyte apheresis device Adacolumn, which selectively depletes activated neutrophils in the colonic microvasculature, thereby reducing mucosal inflammation [52].

Beyond neutrophils, ILCs play critical roles in antimicrobial defense, tissue repair, and mucosal homeostasis. ILCs are classified into three subsets: ILC1s, which produce interferon‐γ and IL‐18 [53]; ILC2s, which secrete IL‐5 and IL‐13 [54]; and ILC3s, characterized by production of IL‐22, IL‐17 and GM‐CSF [55]. In IBD, altered ILC profiles are frequently observed, notably an increase in ILC1s and ILC2s and a concomitant reduction in protective ILC3s within inflamed tissues [56, 57, 58, 59, 60]. Under normal circumstances, ILC3s contribute to mucosal integrity through IL‐22 secretion [61, 62] but dysregulation, especially excessive IL‐22 production, may paradoxically exacerbate inflammation by recruiting neutrophils and compromising the epithelial barrier [63, 64]. Importantly, microbial signals mediated via receptors such as FFAR2 and the aryl hydrocarbon receptor (AhR) regulate ILC3 activity and thereby influence colitis severity in experimental models [61, 62, 69]. Notably, patients with mild to moderate IBD, and particularly those with CD, often exhibit elevated IL‐22 levels, which correlate positively with disease activity [65, 66]. Dysbiosis may trigger excessive IL‐22 secretion by Th17 cells, resulting in aberrant epithelial proliferation, dysplasia, severe colitis [67], and ultimately complications such as anal fistula formation [68, 69]. Thus, therapeutic modulation of ILCs holds promise, though it demands careful calibration to preserve intestinal homeostasis.

Macrophages are also central to the IBD‐associated inflammatory milieu. In response to PAMPs and DAMPs, recruited macrophages release proinflammatory cytokines such as TNF‐α and IL‐1β [7970, 71, 72]. In animal models of colitis, macrophage recruitment via CCR2–CCL2 signaling is critical for disease development [70, 73]. Under homeostatic conditions, colonic macrophages typically express CX3CR1 and Major Histocompatibility Complex (MHC) II, exhibit robust phagocytic activity, low responsiveness to Toll‐like receptor (TLR) stimulation, and constitutively produce IL‐10 [70], thereby contributing to mucosal tolerance. However, in IBD, this balance is disrupted: macrophages can participate both in formation of inflammatory tertiary lymphoid structures and in generating anti‐inflammatory IgA responses [74]. Genetic polymorphisms in CX3CR1 or its ligand CX3CL1 have been associated with IBD phenotypes in patients [75], though their functional impact remains ambiguous, as experimental deficiency has either ameliorated [76] or worsened [77] colitis in different animal studies. Importantly, signaling through the IL‐10 receptor (IL‐10R) is essential for anti‐inflammatory macrophage functions and mucosal healing [78, 79, 80]. Indeed, mice lacking IL‐10R in macrophages develop spontaneous, severe colitis reminiscent of pediatric IBD [80], and macrophage‐derived IL‐10 has been shown to support epithelial regeneration via the CREB/WISP‐ axis [81, 82]. In addition, M2‐like macrophages that are differentiated under the influence of IL‐33 via STAT6 promote epithelial repair through activation of WNT signaling [83, 84].

Yet macrophages can also contribute to long‐term complications. Aberrant activation, characterized by secretion of TGF‐β1, CTGF, and fibroblast activation protein, can drive fibrosis, a common IBD complication [85]. In this context, macrophages may promote a macrophage‐to‐myofibroblast transition, leading to increased myofibroblast accumulation and extracellular matrix deposition [86, 87].

Finally, DCs located in Peyer's patches, lymphoid follicles, and throughout gut tissues constantly sample luminal antigens [88, 89, 90], playing a central role in maintaining homeostasis. Subsets such as conventional DC1s (cDC1s) and CD103^+^ cDC2s support tolerance to commensal microbiota by promoting regulatory T (Treg) cell induction and IL‐10 production [91, 92, 93]. Loss of these protective DC subsets increases vulnerability to colitis in experimental models [91, 93]. In IBD, however, colonic DCs often display immature phenotypes with altered receptor expression [94, 95, 96] and reduced representation of beneficial subsets [97]. Dysbiosis can trigger aberrant DC activation, prompting production of proinflammatory cytokines including IL‐12, IL‐6, and IL‐18, which in turn foster Th1 responses and exacerbate inflammation [98]. Moreover, plasmacytoid DCs may contribute to chronicity by migrating to secondary lymphoid organs and producing Th1‐associated cytokines [99]. While the role of CD103^+^ cDC2s remains somewhat uncertain, studies indicate that IRF4^+^ cDC2s facilitate early T‐cell priming [100], whereas cDC1s appear to exert protective functions: their absence aggravates colitis [101].

With regards to differences in innate immune responses between patients with UC versus CD, one study by applying multidimensional single‐cell analytics using mass cytometry (CyTOF) revealed mucosal and blood immunologic signatures that differentiate UC from CD [102]. The colonic mucosa from patients with UC is characterized by expansion of HLA‐DR^+^CD56^+^ granulocytes and reductions in ILC3 cells. High IL1β^+^ DCs and IL1β^+^ plasmacytoid DCs were observed in colonic mucosa from patients with active CD. Peripheral blood mononuclear cells from patients with active CD showed increased levels of IL1β^+^ DCs and IL1β^+^ plasmacytoid DCs, IL1β^+^ monocytes, and fewer ILC1 cells with respect to UC patients. These data identify IL1β^+^ signatures specific to CD, suggesting that targeting IL1β^+^ may be a promising strategy in a subgroup of CD patients [102].

Another study, that analyzed RNA‐seq datasets from 26 independent studies, reported a specific imbalance of the immune system not only disease‐specific (CD versus UC) but also across different intestinal segments (ileum, colon, and rectum) [103]. In the ileum, the increase in innate immune cells was more pronounced in CD than UC. In contrast, resting NK cells, monocytes, activated DCs, and resting mast cells were all increased in the rectum of UC, compared with CD [103].

#### 2.1.2 Intestinal Adaptive Immune System

Adaptive immunity plays a pivotal role in the pathogenesis of IBD, functioning in tandem with innate immune mechanisms to amplify and sustain intestinal inflammation. It is widely accepted that adaptive immune responses in IBD emerge secondary to defects in innate immunity, such as compromised epithelial barrier integrity or impaired microbial sensing. These downstream responses, shaped by distinct genetic susceptibilities, underlie the heterogeneous clinical and immunological phenotypes observed in IBD.

B lymphocytes contribute to disease pathophysiology through the production of antibodies directed against microbial antigens, including Saccharomyces cerevisiae, Escherichia coli outer membrane protein C, and bacterial flagellin. Although these antibodies are frequently detected in IBD patients, their precise pathogenic role remains uncertain. Early clinical trials in UC using anti‐CD20 monoclonal antibodies such as rituximab yielded disappointing outcomes, initially suggesting minimal involvement of B cells [104]. However, subsequent findings revealed that rituximab fails to deplete tissue‐resident antibody‐secreting plasma cells, which lack high CD20 expression [105], indicating a potential failure in targeting relevant B cell subsets.

Inflamed intestinal tissues in IBD are characterized by a shift from protective IgA dominance toward IgG predominance [106, 107]. Experimental models show that impaired IgA responses promote dysbiosis and intestinal inflammation [108]. In UC, elevated anticommensal IgG within the colonic mucosa drives inflammation via complement activation, neutrophil recruitment, and activation of macrophages and Type 17 immune responses [109]. These findings suggest that therapeutic strategies aimed at depleting IgG‐producing plasma cells or restoring IgA predominance may hold promise. However, further investigation is needed to delineate the contributions of distinct B cell subsets in disease pathogenesis and treatment response.

T lymphocytes, primed by DCs and guided by mucosal homing receptors such as CCR9, CCR10, and integrins α4β7 and αEβ7, infiltrate intestinal tissues and differentiate into effector, regulatory, or memory phenotypes [110, 111]. Effector T cells secrete proinflammatory cytokines that mediate microbial defense, Treg cells suppress immune activation, and memory T cells ensure long‐term immunity [112]. Among CD4^+^ effector cells, T helper 1 (Th1) and T helper 17 (Th17) subsets, that produce IFN‐γ and IL‐17A, IL‐17F, and IL‐22, respectively, have been implicated in both murine and human IBD [113, 114].

Treg cells, defined by expression of the transcription factor FOXP3, are critical for maintaining mucosal tolerance. They exert immunoregulatory functions through inhibitory molecules such as CTLA‐4 and TIGIT and the production of IL‐10 and TGF‐β. Unexpectedly, increased numbers of Treg cells have been observed in inflamed IBD tissues [115], potentially reflecting functional impairment or transient FOXP3 expression. Notably, many studies do not account for the heterogeneity of Treg cells, leaving open questions regarding subset‐specific functions in IBD. Specific Treg subsets in CD and UC have been reported to produce IL‐17A and IFN‐γ while retaining suppressive capacity, raising important questions about Treg cell plasticity and stability [102, 116].

Tissue‐resident memory T (TRM) cells (CD4^+^/CD8^+^ CD69^+^CD103^+^) are enriched in inflamed mucosa of UC and CD patients, where they contribute to chronic inflammation and epithelial barrier disruption [117, 118]. Recent findings indicate that TRM cells can exit tissues and re‐enter systemic circulation. In UC, circulating CD8^+^ T cells have been shown to share clonal identity with mucosal TRM cells during active disease [119, 120], suggesting that recirculating TRM cells may also play a role in extraintestinal manifestations.

Current IBD therapies primarily target cytokines produced by Th1 and Th17 cells or interfere with lymphocyte trafficking by blocking integrin‐mediated homing (see Section 3). In parallel, therapeutic strategies aimed at restoring Treg function are being actively explored. These include microbiota‐based interventions to promote Treg differentiation, low‐dose IL‐2 therapy selectively expanding Treg cells over effector T cells [121], IL‐2–anti‐IL‐2 immune complexes [122], engineered IL‐2 muteins [123], and adoptive transfer of ex vivo‐expanded peripheral Treg cells [124]. Despite progress, a more refined understanding of Treg heterogeneity is essential to exploit their therapeutic potential without compromising intestinal homeostasis.

The two principal forms of IBD share many pathogenic elements but diverge in immune polarization, anatomical distribution, and tissue‐injury pattern, with direct consequences on therapeutic intervention. UC is characterized by continuous, mucosa‐restricted inflammation of the colon dominated by a Th2‐like cytokine profile featuring IL‐13, IL‐5, and atypical Th17 responses; the inflammatory infiltrate remains largely superficial, sparing the muscularis. CD, in contrast, exhibits discontinuous, transmural inflammation that can affect any segment of the gastrointestinal tract, with a Th1/Th17 dominance, high expression of IFN‐γ, IL‐12, and IL‐23, and a pathogenic Th17 signature (IL23R^+^, GZMB^+^, IL‐18RAP^+^) that is enriched in mesenteric lymph nodes of CD relative to UC [125]. Granulomas, fissuring ulcers, fibrosis, and stricturing–fistulizing complications are CD‐specific manifestations of this transmural, granulomatous biology.

These pathophysiological differences could explain key asymmetries in therapeutic response. Anti‐IL‐12/23 (ustekinumab) and anti‐IL‐23p19 (risankizumab, mirikizumab) agents are effective in both UC and CD because they target the central IL‐23/Th17 axis that operates in both diseases, albeit more prominently in CD. Conversely, selective anti‐IL‐17A (secukinumab) and anti‐IL‐17RA (brodalumab) antibodies, despite a strong mechanistic rationale, paradoxically worsened CD in randomized trials and have led to new‐onset IBD cases in psoriasis cohorts treated with IL‐17 inhibitors, a phenomenon attributed to the indispensable barrier‐protective role of IL‐17A in intestinal epithelium [126].

Recent CyTOF analysis of colonic mucosa from IBD patients may clarify why IL‐17A blockade is less effective in CD. The study identified a mucosal cytokine axis that can help distinguish UC from CD: IL17A‐producing T cells were expanded in UC, whereas IFN‐γ^+^ TNF^+^ T cells were expanded in CD. Specifically, UC mucosa showed increased IL17A^+^ CD161^+^ effector‐memory T cells and IL17A^+^ Treg cells, while CD mucosa exhibited higher proportions of IL‐1β^+^ HLA‐DR^+^ CD38^+^ T cells and IL‐1β^+^ TNF^+^ IFN‐γ^+^ naïve B cells [102].

Anti‐α4β7 integrin (vedolizumab) has shown greater absolute efficacy in UC than in CD, consistent with the gut‐selective trafficking pattern of UC effector lymphocytes; anti‐α4 (natalizumab) is conversely active in CD but carries CNS risks. JAK inhibitors (tofacitinib, upadacitinib, filgotinib) and S1P‐receptor modulators (ozanimod, etrasimod) are predominantly active in UC, whereas upadacitinib has recently extended JAK efficacy into CD. The 2024 PROFILE trial demonstrated that newly diagnosed CD patients benefit substantially from a top‐down strategy of early combination immunosuppression with infliximab plus immunomodulator over an accelerated step‐up approach, irrespective of the prognostic T‐cell biomarker subgroup [127]. Notably, the negative biomarker‐stratification arm of PROFILE underscores how easily an apparently promising single‐biomarker assay can fail to predict clinical benefit when tested in an adequately powered prospective trial, a cautionary lesson directly relevant to the precision‐medicine framework.

Other recent data reveal immune imbalances that are not only disease‐specific (CD versus UC) but vary also by intestinal segment (ileum, colon, rectum). For instance, UC patients show higher B‐cell levels in the rectum but lower B‐cell levels in the ileum compared with CD patients. T‐cell counts are increased in the rectum of both diseases, although the increase is more pronounced in UC than in CD [103]. More specifically, UC rectal mucosa exhibited significant increases in CD8^+^ T cells, resting memory CD4^+^ T cells, and activated memory CD4^+^ T cells relative to CD, whereas Treg cells were significantly reduced in UC compared with CD.

### 2.2 Genetic Predisposition

Early studies revealed a strong heritable component in IBD, particularly in CD, with elevated risk among first‐degree relatives [128, 129, 130, 131, 132]. Genome‐wide association studies have identified over 240 risk loci affecting microbial sensing (e.g., **NOD2**), autophagy (**ATG16L1**), epithelial barrier genes (**ECM1**), and immune pathways (**IL23R**, **IL10**) [128, 129, 130, 131, 132]. However, these variants explain only 8–13% of CD and 4–7% of UC heritability [133]. Notably, in children with very‐early‐onset IBD, monogenic defects, especially in IL‐10 signaling, may have a more prominent role [134]. Recent genetic advances continue to uncover key regulators of mucosal homeostasis and IBD pathogenesis.

Among these, epigenetic modifications have emerged as critical mediators linking genetic susceptibility to environmental exposures. Epigenomic profiling has revealed that DNA methylation and miRNA signatures are altered in IBD, with distinct patterns correlating with disease susceptibility, activity, behavior, and colorectal cancer (CRC) risk [135, 136, 137, 138, 139, 140, 141]. Several DNA methylation‐ and miRNA‐based panels have shown clinical utility in diagnosis, disease stratification, and CRC surveillance, offering high sensitivity and specificity. Beyond biomarker potential, epigenetic regulation has been implicated in essential IBD‐related processes, including immune modulation, intestinal barrier integrity, and autophagy. Functional studies have demonstrated involvement of epigenetic changes in T‐cell differentiation [142, 143], IL‐23/Th17 [144] and STAT3 signaling [145, 146], and epithelial permeability [147, 148], further enriching our understanding of the immunopathology of IBD. These insights suggest promising avenues for therapeutic modulation of epigenetic targets in clinical settings.

Concurrently, genome‐wide profiling has revealed molecular features, including gene expression and epigenetic changes, that distinguish IBD from healthy states and differentiate CD and UC subtypes. In colonic tissue, two molecular CD subtypes have been identified, differing in metabolism and immune signaling [128]. Other studies have pinpointed at genes overexpressed in IBD tissue, such as oncostatin M, predictive of anti‐TNF therapy failure [149]. However, cell‐type heterogeneity in whole‐tissue samples limits resolution, as transcriptomic signals often reflect dominant cell populations without clear cellular origin.

Advances in single‐cell RNA sequencing and mass cytometry have revealed IBD‐specific signatures and uncovered novel fibroblast, epithelial, and immune cell subsets enriched or depleted in disease [102, 106, 107, 150, 151, 152]. For instance, the GIMATS cellular module, encompassing IgG‐producing plasma cells, inflammatory mononuclear phagocytes, activated T cells, and stromal cells, was found to be enriched in a subgroup of patients with ileal CD and associated with a lack of durable remission following anti‐TNF therapy [106]. Distinct genetic signatures have been found for UC and CD. For example, CARD15/NOD2 mutations, functionally linked to a deficient antimicrobial defense, are significantly associated with risk of CD and not with UC [153]. Large‐scaled high‐density mapping of the MHC region evidenced the equivalent contribution of Class I and Class II HLA variants to disease risk in CD, while HLA Class II variation has a more important role in UC [154].

More recently, using the Immuno‐chip platform designed to map loci of immune‐mediated disease, composite genetic scores derived from 163 independent risk loci were able to distinguish all CD subtypes (ileal and colonic) from UC [155]. Interestingly, these findings remained significant even after excluding variants in NOD2 and the MHC region [155]. This suggests that most IBD risk variants contribute only modestly to disease sub‐phenotype, and that environmental factors (such as diet, microbiota, and smoking) might be important contributors.

Another study used a composite model integrating genetics, phenotypic, and serologic markers to predict time to first surgery in CD more accurately than models relying on clinical data, genetics, or clinical + serology alone. Notably, variation at the IL12B locus showed a consistent association with both the need for surgery and the time to surgery, suggesting IL12B may be a potential target for therapeutic intervention [156]. The therapeutic translation of such genetic signals, however, has been uneven: while anti‐IL‐12/23 strategies validated the IL‐12/23 axis in CD, single‐locus susceptibility variants have so far rarely translated into individual‐patient predictive biomarkers, underscoring the need for composite, multiomic stratification approaches.

Together, genetic, transcriptomic, and epigenomic studies continue to refine our understanding of IBD pathogenesis and hold promise for the identification of cell‐type‐specific biomarkers and therapeutic targets that can inform precision medicine approaches.

### 2.3 Microbiota

Humans host trillions of microbes—bacterial, viral, fungal, and eukaryotic—collectively forming the microbiome at barrier surfaces [157]. The distal ileum and colon house the densest and most diverse bacterial communities. This symbiosis supports health by metabolizing nutrients and producing vitamin K and short‐chain fatty acids (SCFAs), while shaping immune development and preventing pathogen colonization. However, when mucosal integrity is breached, commensals may become pathogenic, eliciting immune responses and driving intestinal inflammation [158].

The intestinal microbiome, a complex multikingdom community comprising bacteria, fungi, and viruses, is inextricably linked to the pathogenesis of IBD. Perturbations in this intricate microbial ecosystem, often referred to as dysbiosis, are a hallmark of IBD, with global incidence trends paralleling changes in environmental factors that affect the microbiome.

In IBD, there are characteristic fluctuations in gut bacterial composition and diversity, typically manifesting as an increased ratio of potentially pathogenic to beneficial bacteria and a reduction in overall diversity. Specific bacterial groups like *Enterobacteriaceae*, *Fusobacterium spp*., and *Ruminococcus gnavus* are frequently overrepresented in active IBD, while beneficial species such as *Faecalibacterium prausnitzii* and *Roseburia spp*., crucial producers of SCFAs with anti‐inflammatory properties, are often diminished [159, 160, 161]. The gut mycobiome also undergoes significant changes, with species from genera including *Candida*, *Saccharomyces*, and *Malassezia* frequently altered or enriched. Notably, highly damaging *Candida albicans* strains that produce the toxin candidalysin have been identified in UC patients, contributing to intestinal inflammation by damaging epithelial membranes and activating inflammatory pathways [162]. Furthermore, the gut virome shows disease‐specific alterations, with an increase in both eukaryotic viruses and bacteriophage diversity in CD and UC, including elevated levels of *Caudovirales* members such as *Siphoviridae*, *Myoviridae*, and *Podoviridae* [163].

The impact of the microbiota on IBD pathogenesis is profoundly mediated through its metabolites and its intricate interactions with the host's immune and neuroimmune systems. Microbiota‐derived SCFAs like butyrate and propionate are vital for maintaining intestinal homeostasis, facilitating immune responses, and mitigating inflammation, but their production is often reduced in IBD due to the depletion of beneficial bacteria [164]. Bile acids also play a crucial modulatory role; bacteria‐mediated modifications generate secondary bile acids that exert effects on metabolism and immune responses. For example, levels of Th17‐modulating bile acids, such as 3‐oxolithocholic acid and iso‐lithocholic acid, are decreased in CD, correlating with upregulated TH17‐associated genes [165]. Additionally, novel amino acid‐conjugated bile acids, produced by bacterial genera including *Clostridium*, *Bifidobacterium*, and *Enterococcus*, are enriched in CD patients and can stimulate the farnesoid X receptor receptor [166]. Beyond these, tryptophan metabolites, such as indolamines produced by bacteria like *Morganella morganii*, have been found to induce DNA damage and increase intestinal permeability, thereby exacerbating inflammation [167].

From an immune perspective, human genetic studies highlight the critical role of innate immunity, with IBD risk variants in genes such as *CARD9*, *IL23R*, and autophagy‐related genes like *ATG16L1* being strongly associated with compromised antimicrobial defenses and barrier integrity [168, 169]. These genetic predispositions often manifest as defective immune responses to specific microbial components or impaired Paneth cell function. The Th17 response, frequently directed against commensal fungi like *C. albicans*, represents a significant adaptive immune mechanism [170]. Indeed, commensal *Candida albicans* enhances systemic immunity by promoting IL‐17‐driven Th17 and neutrophil responses, offering protection against extracellular pathogens like *S. aureus*. However, this benefit is counterbalanced by increased risk of airway inflammation [170]. Moreover, both bacteria‐reactive and fungi‐specific B cells and cytotoxic T cells contribute to IBD pathology, while secretory IgA (sIgA) produced by B cells is essential for maintaining gut homeostasis and can target fungal hyphae [171]. The neuroimmune system also demonstrates a crucial crosstalk with the gut microbiota, which directly modulates enteric‐associated neurons, thereby influencing gut motility and pain perception. SCFAs, for instance, can reduce neuronal activity [172], while dysregulation of serotonin, modulated by bacteria like *Ruminococcus gnavus*, contributes to altered gastrointestinal transit and colonic secretion observed in irritable bowel syndrome and potentially IBD patients [173]. Conversely, disruption of nociceptors, specialized pain‐sensing neurons, has been shown to alter microbiota composition and exacerbate intestinal inflammation [174].

Some studies focused on highlighting differences in bacteriome, mycobiome, and virome between UC and CD. For example, reduced alpha‐diversity bacteriome was observed in a Japanese cohort of UC and CD patients, compared with controls, but the difference was significantly lower in CD than UC [175]. Furthermore, the application of specific analysis to identify ESKAPE pathogens, revealed a significant increase of *Klebsiella pneumoniae*, *Staphylococcus aureus*, *Acinetobacter baumannii*, and *Pseudomonas aeruginosa* in CD, but not UC, indicating increased levels of multidrug‐resistant bacteria in CD than in UC patients. In addition, 12 virulent factors, mediating adhesion to and invasion of intestinal epithelial cells in *Escherichia coli*, were significantly enriched in CD suggesting the implementation of a therapeutic approach targeting Adherent‐Invasive *Escherichia coli* in patients with CD. Mycobiome signatures also differs between UC and CD. *Saccharomyces cerevisiae* and *Debayomyces hansenii* were enriched in CD, whereas *Saccharomyces paradoxus* and *Saccharomyces kudriavzevii* were enriched in UC. A significant depletion of *Exophiala mesophila* was noted only in UC [175]. Interestingly, the taxonomic differences observed between UC and CD patients resulted in different microbiome functionality. A recent study indeed reports that CD patients are significantly deficient in pathways for the production of SCFAs that have important functions to maintain immune homeostasis and cellular membrane integrity [176].

This multifaceted understanding of the microbiota's role in IBD offers unparalleled opportunities for novel diagnostic and therapeutic strategies. Dietary interventions, ranging from anti‐inflammatory diets and exclusive enteral nutrition to the targeted modulation of specific dietary components like fermented foods, demonstrably alter the microbiome and can reduce inflammation or induce remission, especially in pediatric CD [177]. Beyond these, advanced biotechnological approaches are under development, including bacteriophages [178] designed to precisely target specific pathogenic bacteria and rationally defined bacterial consortia (US National Library of Medicine. *ClinicalTrials.gov* https://Clinicaltrials.Gov/Ct2/Show/NCT05370885, 2024) or engineered probiotic yeasts aiming to suppress inflammation and dysbiosis [179]. Finally, the microbiota serves as a rich source of microbial biomarkers, offering the potential to predict disease course, relapse, and critically, individual responses to existing biological therapies such as anti‐TNF or anti‐integrin agents, thereby paving the way for personalized medicine in IBD management [180].

Although no causal link has been established between microbiome alterations and IBD, the potential for microbiota‐based treatments, such as FMT, has been proposed (see Section 3.2).

Given the numerous factors that contribute to IBD, a key to the success of these molecular subtyping studies could be the simultaneous interrogation of genetic, microbial, and multiple molecular features within a cohort of clinically well‐phenotyped patients. An integrated approach is better‐positioned to capture the landscape of contributing factors and the interactions among them, thereby improving our understanding of IBD pathogenesis. Equally important is the inclusion of prospective and retrospective longitudinal studies, which allow connections to be drawn between intestinal states at an initial time point and their subsequent effects on disease course and response to treatments.

## 3 Current and Emerging Therapies for IBD

The therapeutic landscape for IBD has evolved substantially, driven by advancements in our understanding of the molecular mechanisms underlying intestinal inflammation. This paragraph provides a comprehensive summary of the current treatments for IBD and introduces emerging therapies under development. Patients with IBD often require lifelong medication to modulate the course of the disease, therapeutic goals follow a step‐wise progression starting from symptom control and normalization of inflammatory markers, to histological remission, endoscopic healing, absence of disability, and restoration of quality of life. Current treatments primarily aim to inhibit immune activity by targeting specific inflammatory cytokines. However, a substantial gap remains in strategies that directly promote mucosal regeneration and restore intestinal homeostasis.

### 3.1 Current Therapies

Biologics, essentially antibodies blocking specific molecules with inflammatory activity or involved in immune response activation, are currently the main therapeutic option for IBD. The largest class of biologics are agents targeting TNF‐α, a proinflammatory cytokine found in increased concentrations in the blood, stool, and colonic mucosa of patients with IBD [181].

TNF‐α is predominantly secreted by monocytes, macrophages, and natural killer cells and is a key mediator in the regulation of several inflammatory pathways including the cyclooxygenase‐2 (COX‐2) and inducible nitric oxide synthase (iNOS) pathways [182]. In the 1990s, infliximab, the first anti‐TNF‐α antibody applied to treat IBD, dramatically improved clinical outcomes, particularly in moderate/severe IBD patients. However, clinical remission was observed in only 25% of patients with CD [183] and in 35% of patients with UC after 54 weeks of infliximab treatment [184]. Subsequently, biosimilars targeting TNF‐α, including adalimumab [185, 186, 187], golimumab [188], and certolizumab [189], have been introduced. Yet, they still maintain a limited efficacy since still many patients are not responsive, or lose response, or experience severe side effects to anti‐TNF therapy, such as increased infection rates and new‐onset psoriasiform dermatitis lesions (Figure 2).

Numerous new biologics are currently approved as therapies for CD or UC patients or are in Phase III clinical trials (see Table 1).

**TABLE 1:** Immune‐targeted therapies approved or in late‐stage development for inflammatory bowel disease.

Drug	Molecule type	Target	Pathology indication	Delivery route	Pivotal trials (registration no. at www.clinicaltrials.gov)	Trial phase	Study duration (years)	Authors conclusions	References
Secukinumab	Human monoclonal antibody	IL‐17A	Moderate to severe Crohn's disease	iv	NCT01009281	Phase IIa	2008–2010	Secukinumab therapy failed to reduce mean CDAI by ≥50 points more than placebo at Week 6, missing the primary end point. Compared with placebo, secukinumab had a smaller effect and patients on secukinumab showed higher rates of adverse events.	[126]
Infliximab	Chimeric monoclonal antibody	TNFα	CD	iv	ACCENT I	Phase III	1999–2000	Patients with Crohn's disease who respond to an initial dose of infliximab are more likely to be in remission at Weeks 30 and 54, to discontinue corticosteroids, and to maintain their response for a longer period of time if infliximab therapy is maintained every 8 weeks. Maintenance infliximab treatment was safe and well tolerated	[183]
			UC	iv	ACT 1 and ACT 2 (NCT00036439, NCT00096655)	Phase III	2002–2005	An induction regimen of three doses of infliximab followed by maintenance infusions every 8 weeks in patients with moderate‐to‐severe active ulcerative colitis was superior to placebo in achieving clinical response and remission, mucosal healing, and corticosteroid‐sparing effects during 30–54 weeks of therapy.	[184]
Adalimumab	Human monoclonal antibody	TNFα	CD	sc	CLASSIC II	Phase II	2002–2005	Subcutaneous administration of adalimumab resulted in maintenance of remission and response, potential steroid sparing effects, and improved quality of life over 1 year in infliximab‐naive Crohn's disease patients with moderate to severe disease activity compared with placebo.	[185]
			Moderate to severe Crohn's disease	sc	CHARM	Phase III	2003–2005	Adalimumab 40 mg every other week and 40 mg weekly are more effective than placebo in maintaining clinical remission and response in patients with moderate to severe CD through 56 weeks. Adalimumab is also effective for maintaining corticosteroid‐free remission and completely closing fistulas.	[186]
			Moderately to severely active UC	sc	ULTRA 1, 2, 3	Phase III	2006–2013	Adalimumab treatment for up to 4 years is well tolerated and is beneficial for patients with moderately to severely active UC in maintaining remission and mucosal healing. The improvement in quality of life, work productivity, and low hospitalization and colectomy rates support the benefit of long‐term adalimumab therapy in a patient population who failed conventional therapy and/or previous anti‐TNF therapy. Read collectively, these adalimumab data reflect a broader principle of contemporary IBD management: the most clinically meaningful benefits of any biologic class are seen when the agent is positioned not as last‐line salvage but as part of an early, mechanism‐matched strategy in patients selected by disease behavior and prior treatment exposure.	[187]
Golimumab	Human monoclonal antibody	TNFα	Adults with moderate‐to‐severe active ulcerative colitis	sc	PURSUIT (NCT00488631)	Phase III	2007–2011	100‐mg golimumab dose achieved long‐term clinical remission and mucosal healing at both Weeks 30 and 54, in addition to showing positive trends for maintenance of clinical remission through 1 year.	[188]
Certolizumab pegol	Pegylated humanized Fab' antibody fragment	TNFα	Adults with moderate‐to‐severe Crohn's disease	sc	PRECISE 2 (NCT00152425)	Phase III	2004–2005	Patients with moderate‐to‐severe active Crohn's disease who had a response to induction therapy with certolizumab pegol and continued to receive certolizumab pegol as maintenance therapy were more likely to have a maintained response and remission at 26 weeks than were those who switched to placebo.	[189]
Ustekinumab	Human monoclonal antibody	p40 subunit of IL‐12 and IL‐23	Patients with moderately to severely active Crohn's disease refractory to treatment with TNF antagonists	iv induction therapy (UNITI 1, 2) sc maintenance therapy (IM‐UNITI)	UNITI 1, 2 (NCT01369329, NCT01369342) IM‐UNITI (NCT01369355)	Phase III	2011–2015	Intravenous ustekinumab induces response and remission in patients with moderately to severely active Crohn's disease that is refractory to either TNF antagonists or conventional therapy. Among patients who had a response to intravenous induction, subcutaneous ustekinumab administered at a dose of 90 mg every 8 weeks or every 12 weeks was more effective than placebo for maintaining remission.	[190, 193, 194]
			Patients with moderate‐to‐severe ulcerative colitis with inadequate response to or unacceptable side effects from TNF antagonists, vedolizumab, or conventional (i.e., nonbiologic) therapy	iv induction therapy sc maintenance therapy	UNIFI (NCT02407236)	Phase III	2015–2018	In patients with moderate‐to‐severe ulcerative colitis, despite current or previous treatment with conventional or biologic therapy, ustekinumab was more effective than placebo for inducing and maintaining remission.	[192]
Risankizumab	Humanized monoclonal antibody	p19 subunit of IL‐23	Moderate‐to‐severe Crohn's disease, with inadequate response to one or more approved biologics or conventional therapy (ADVANCE) or to biologics (MOTIVATE)	iv	ADVANCE (NCT03105128) and MOTIVATE (NCT03104413)	Phase III	2017–2020	These trials demonstrated that risankizumab is a safe and effective treatment for inducing clinical remission and endoscopic response in patients with Crohn's disease who have not responded to previous conventional or biologic therapies.	[195]
	Humanized monoclonal antibody	p19 subunit of IL‐23	Moderate‐to‐severe Crohn's disease, with inadequate response to one or more approved biologics or conventional therapy	iv induction therapy (ADVANCE, MOTIVATE) sc maintenance therapy (FORTIFY)	FORTIFY (NCT03105102)	Phase III	2018–2020	Subcutaneous risankizumab is a safe and efficacious treatment for maintenance of remission in patients with moderately to severely active Crohn's disease. Greater clinical remission and endoscopic response rates were reached with 360 mg risankizumab versus placebo.	[196]
	Humanized monoclonal antibody	p19 subunit of IL‐23	Moderate‐to‐severe Crohn's disease	iv induction therapy (ADVANCE, MOTIVATE) sc maintenance therapy (FORTIFY)	FORTIFY‐OLE (NCT03105102)	Phase III	Ongoing	Data presented at 2 years of maintenance treatment show durable efficacy with clinical and endoscopic improvements by Week 152.	[197, 198]
	Humanized monoclonal antibody	p19 subunit of IL‐23	Moderately to severely active ulcerative colitis. Patients from the induction trial with an adequate clinical response	iv induction therapy (at Weeks 0, 4, and 8)sc maintenance therapy (every 8 weeks for 52 weeks)	INSPIRE (NCT03398148) and COMMAND (NCT03398135)	Phase III	2018–2023	Risankizumab improved clinical remission rates in an induction trial and in a maintenance trial in patients with moderately to severely active ulcerative colitis.	[199, 206]
Mirikizumab	Humanized immunoglobulin G4–monoclonal antibody	p19 subunit of IL‐23	Moderate to severely active UC with inadequate response to conventional and advanced therapies. In maintenance study, patients who had a clinical response to mirikizumab therapy at Week 12	iv induction therapy (every 4 weeks) sc maintenance therapy (every 4 weeks for an additional 40 weeks)	LUCENT‐1 (NCT03518086), LUCENT‐2 (NCT03524092)	Phase III	2018–2021	Mirikizumab has demonstrated sustainable efficacy in patients with moderately to severely active UC, in whom conventional or advanced therapies have failed.	[200, 201]
	Humanized immunoglobulin G4–monoclonal antibody	p19 s p19 subunit of IL‐23	Moderate to severely active UC. Patients who had a clinical response to mirikizumab therapy at Week 12	sc continuous therapy (every 4 weeks up to W 104)	LUCENT‐3 (NCT03519945)	Phase III	Ongoing	Long‐term treatment with mirikizumab up to 104 weeks is associated with a sustained and durable effect on clinical response/remission, endoscopic, histologic, symptomatic, and quality‐of life outcomes in patients who had previously failed biologic therapy and those who had not.	[202]
	Humanized immunoglobulin G4–monoclonal antibody	p19 subunit of interleukin‐23	Patients with moderately to severely active Crohn's disease. Maintenance study (VIVID‐2) in endoscopic responders and nonresponders at 1 year	VIVID‐1: iv induction therapy (at weeks (0, 4, and 8) + sc (every 4 weeks for 1 year) VIVID‐2: sc up to W 104	VIVID‐1 (NCT03926130), VIVID‐2 (NCT04232553)	Phase III	Ongoing	High maintenance rates of endoscopic response and remission were seen in moderately to severely active CD patients who responded at 1 year, with more achieving remission in Year 2 of mirikizumab treatment. Over 30% of 1‐year nonresponders gained response after mirikizumab reinduction.	[203, 204]
Natalizumab	Humanized monoclonal antibody	α4 integrin	Moderate‐to‐severe Crohn's disease	iv	ENACT‐1 (NCT00032786) and ENACT‐2 (NCT00032799)	Phase III	2001–2004	Induction therapy with natalizumab for Crohn's disease resulted in small, nonsignificant improvements in response and remission rates. Patients who had a response had significantly increased rates of sustained response and remission if natalizumab was continued every 4 weeks. The benefit of natalizumab will need to be weighed against the risk of serious adverse events, including progressive multifocal leukoencephalopathy.	[211]
Vedolizumab	Humanized immunoglobulin G1 monoclonal antibody	α4β7 integrin	Moderately to severely active Crohn's disease in whom conventional therapy failed	iv	GEMINI‐2 (NCT00783692)	Phase III	2008–2012	Modest effect of vedolizumab on the induction of clinical remission and its nonsignificant effect on the CDAI‐100 response at Week 6. Among patients who had a response to vedolizumab at Week 6, the rates of clinical remission at Week 52 were significantly higher among patients receiving vedolizumab every 8 weeks or every 4 weeks than among those receiving placebo. During the maintenance phase, serious infections occurred in 5.5% of the patients receiving vedolizumab, as compared with 3.0% of the patients receiving placebo.	[212]
			CD (GEMINI 2 and GEMINI 3) and UC (GEMINI 1) patients	iv	GEMINI 1 (NCT00783718); GEMINI 2 (NCT00783692); GEMINI 3 (NCT01224171)	Phase III	2008–2012	Patients who received vedolizumab during the maintenance phase of GEMINI 2 were 45% less likely to develop new arthritis/arthralgia than patients who received placebo [HR, 0.55; 95% CI, 0.36–0.84] and 37% less likely to develop either new/worsening arthritis/arthralgia [HR, 0.63; 95% CI, 0.44–0.89]. In GEMINI 2, clinical response and remission at Weeks 6 and 52 were associated with sustained resolution of arthritis/arthralgia. These effects were not evident in GEMINI 3. In conclusion, analyses of the GEMINI studies indicate that long‐term treatment with vedolizumab is not associated with new occurrences or worsening of arthritis/arthralgia in UC patients and suggest potential benefits in reducing the likelihood of new occurrences or worsening of these events in CD patients even after corticosteroid withdrawal.	[213]
			Patients with active CD and patients with active UC	iv	OBSERV‐IBD	Phase IV	2014	A potential benefit of vedolizumab therapy in the management of patients with IBD and associated inflammatory arthralgia/arthritis has been observed in the present study especially in patients that achieved complete remission of IBD. However, such benefit may be limited to patients with inflammatory arthralgia/arthritis that runs in parallel with intestinal activity. Paradoxical cutaneous manifestation may occur upon vedolizumab therapy suggesting that paradoxical inflammation is not restricted to the anti‐TNF drug class.	[214]
Tofacitinib	Janus kinases (JAK) inhibitor	Janus kinases 1, 3	Patients with moderately to severely active UC	Oral (10 mg twice daily) induction therapy for 8 weeks) Oral (5 mg or 10 mg twice daily) maintenance therapy (for 52 weeks)	OCTAVE induction 1 (NCT01465763), induction 2 (NCT01458951), OCTAVE sustain NCT01458574()	Phase III	2012–2016	At 8 weeks, higher rates of remission, of mucosal healing and clinical response in patients treated with tofacitinib than with placebo. Efficacy with respect to these end points was maintained at 52 weeks.	[217]
Upadacitinib	JAK inhibitor	Janus kinase 1	Patients with moderately to severely active UC with inadequate response, loss of response or intolerance to corticosteroids, immunosuppressive agents, and/or biologics	os	U‐ACHIEVE induction (NCT02819635); U‐ACCOMPLISH (NCT03653026) and (U‐ACHIEVE maintenance (NCT02819635)	Phase III	2018–2021	In patients with moderately to severely active UC, upadacitinib treatment achieves complete symptom resolution and endoscopic remission, as early as Week 8 of induction treatment, with these benefits maintained long‐term after 1 year of maintenance therapy.	[220]
Filgotinib	JAK inhibitor	Janus kinase 1	Moderately to severely active ulcerative colitis	os	SELECTION (NCT02914522)	Phase IIa–Phase III	2016–2020	Clinical remission was not significantly different between filgotinib 100 mg and placebo at Week 10, but was significant by Week 58. In the induction studies, serious adverse events had incidences similar between treatment groups. In the maintenance study, serious adverse events were reported in 4·5% patients given filgotinib 100 mg, (7·7% patients in the respective placebo group, 4·5% patients in the filgotinib 200 mg group, and no patients in the respective placebo group.	[221]
			Patients with moderate‐to‐severe Crohn's disease	os	Induction trials: U‐EXCEL (NCT03345849) and U‐EXCEED (NCT03345836) Maintenance trial: U‐ENDURE (NCT03345823)	Phase III	2017–2022	A significantly higher percentage of patients who received upadacitinib than those who received placebo had clinical remission and an endoscopic response, either at Week 12 and at Week 52.	[222]
			Moderately to severely active ulcerative colitis	os	DIVERSITY (NCT02914561)	Phase III	2016–2020	In CD patients, filgotinib 200 mg did not achieves clinical remission and an endoscopic response at Week 10, but it showed efficacy at Week 58.	[223]
Ozanimod	S1P receptor modulator	S1P receptor subtypes 1 and 5	Moderately to severely active ulcerative colitis	os (for 10 weeks in the induction therapy; for 52 weeks in the maintenance therapy)	True North (NCT02435992) induction and maintenance trial	Phase III	2015–2020	Treatment with ozanimod led to significant improvements, as compared with placebo, in the incidence of clinical remission and in all key secondary clinical, endoscopic, and histologic end points at Weeks 10 and 52.	[225]
			Moderately to severely active ulcerative colitis (patients who achieved clinical response after 1 year of ozanimod treatment)	os (3 years of continuous ozanimod)	True North open‐label extension [OLE] (NCT02531126)	Phase III	Ongoing	Among patients who achieved clinical response after 1 year of ozanimod treatment during True North trial, a high percentage sustained clinical and mucosal efficacy over 2 additional years in the OLE.	[226]
Etrasimod	S1P receptor modulator	S1P receptor subtypes 1, 4, and 5	Moderately to severely active ulcerative colitis	os (12‐week induction followed by a 40‐week maintenance)	ELEVATE UC 12 (NCT03996369), ELEVATE UC 52 (NCT03945188)	Phase III	2019–2021	Higher rates of clinical remission were observed after induction (Week 12 vs. placebo group) and after maintenance (at Week 52) therapies (vs induction group).	[227]

For each drug, the table reports the type of molecule and molecular target, the IBD indication (UC and/or CD), route and posology of administration, the pivotal randomized clinical trials supporting regulatory approval or current investigational status, and the main efficacy and safety outcomes reported in each trial.

*Abbreviations*: iv, intravenous; sc, subcutaneous; os, oral; CD, Crohn's disease; UC, ulcerative colitis; TNF, tumor necrosis factor; IL, interleukin; JAK inhibitors, Janus kinase inhibitors; S1P receptor, sphingosine‐1‐phosphate receptor.

These biologics block Type 1 and Type 17 immunity (Th1 and Th17 cells) by targeting their cytokine signaling (by using antibodies against IL‐12 and IL‐23, and IL‐17 and IL‐17 receptor) or by inhibiting lymphocyte trafficking (such as anti‐integrins antibodies) (Figure 2).

For example, antibodies, such as ustekinumab, which targets the p40 subunit shared by IL‐12 and IL‐23, have been approved by the United States Food and Drug Administration (US FDA) and European Medicines Agency (EMA) based on demonstrated effectiveness in treating CD and UC [190, 191, 192]. Ustekinumab exerts its effects through dual cytokine blockade; by blocking IL‐12, it inhibits the differentiation of naive CD4^+^ T cells into IFNγ‐producing Type 1 Th1 cells, while by blocking IL‐23, it also inhibits their differentiation into Th17 cells.

Endoscopic and histologic studies involving participants from clinical trials UNITI‐1 [190], UNITI‐2 [190], and IM‐UNITI [190] revealed that ustekinumab led to greater reductions in the Simple Endoscopic Score for CD and the global histologic activity score at Week 8 compared with the placebo [193, 194]. Furthermore, ustekinumab was observed to effectively control psoriasiform skin lesions associated with anti‐TNF‐α treatment. Ustekinumab demonstrated its highest clinical efficacy in CD patients who were anti‐TNF naïve [190]. In the UNIFI trial involving patients with moderate‐to‐severe UC, ustekinumab was shown to be more effective than placebo in achieving both induction and maintenance of clinical remission and histo‐endoscopic mucosal healing [192].

More recently, risankizumab, an antibody selectively targeting the p19 subunit of the IL‐23 receptor, has proven effective in treating CD, both in induction and maintenance phases [195, 196, 197].

At Week 12, patients with CD treated with risankizumab exhibited significantly higher rates of clinical and endoscopic remission compared with those receiving placebo (*p* < 0.001) [195].

Additionally, the FORTIFY trials [196] confirmed the sustained efficacy of risankizumab during the maintenance phase at Week 52, with clinical remission rates of 52 versus 41% and endoscopic response rates of 47 versus 22% when compared with placebo. The ongoing FORTIFY open‐label extension (OLE) study is evaluating the long‐term efficacy and safety of risankizumab in patients with moderate‐to‐severe CD. Data presented at 2 years of maintenance treatment show durable efficacy with clinical and endoscopic improvements by Week 152 [197].

Two Phase III clinical trials showed that risankizumab improved clinical remission rates also in patients with moderately to severely active UC during both the induction and maintenance treatment phases [198, 199].

Other anti‐IL‐23 p19 antibodies have demonstrated efficacy and safety in patients with UC, leading to US FDA approval of mirikizumab and guselkumab and EMA approval of mirikizumab for this indication [200, 201]. More recently, LUCENT‐3 trial demonstrated the ability of continuous treatment with mirikizumab to long‐term (up to 104 weeks) improve clinical response/remission, endoscopic, histologic, symptomatic, and quality of life outcomes in UC patients [202].

Furthermore, the Phase III VIVID‐1 study [203] demonstrated mirikizumab's effectiveness also in patients with moderate‐to‐severe active CD. In this study, patients treated with mirikizumab achieved significantly higher rates of clinical remission (45.4 vs. 19.6%) and endoscopic response (38 vs. 9%) compared with those receiving placebo. The ongoing VIVID‐2 study demonstrates that CD patients who responded after 1 year of mirikizumab treatment sustained both endoscopic response and remission, with more patients achieving endoscopic remission during the second year. Additionally, over 30% of patients who did not respond at 1 year achieved a response following mirikizumab reinduction [204].

Cases of drug hypersensitivity associated with the administration of ustekinumab and anti‐IL23 agents have been reported [205]. Furthermore, anti‐IL‐23 drugs can result in elevated liver enzymes and bilirubin levels [200]; [206]. In contrast to anti IL‐12/IL‐23 antibodies, those against IL‐17A or the IL‐17 receptor subunit IL‐17RA have not demonstrated effectiveness in CD [126] and even caused disease worsening, which prevented their approval for treating patients with IBD. These unexpected outcomes may be attributed to the pleiotropic effects of IL‐17A, which not only induces inflammation but also promotes intestinal epithelial barrier function and repair, acts in an autoregulatory loop to limit Th17‐cell pathogenicity, and provides protection against commensal fungi [207, 208, 209, 210].

Among the anti‐integrin molecules, natalizumab, a monoclonal antibody targeting α4 integrins, prevents leukocyte migration through the vascular wall and access the inflammatory intestinal sites. Natalizumab has demonstrated efficacy in inducing and maintaining clinical remission in patients with CD [211]. However, its use has significantly diminished outside the United States due to the associated risk of progressive multifocal leukoencephalopathy resulting from human papillomavirus 2 reactivation. In contrast, vedolizumab, a monoclonal antibody against α4β7 integrin, has received approval for treating CD based on its ability to induce clinical remission in both patients exposed or not exposed to anti‐TNF‐α therapy, as evidenced by the GEMINI‐2 study [212]. Published data from the posthoc GEMINI study [213] and the prospective OBSERV‐IBD study [214] suggest that vedolizumab may be beneficial for patients with CD exhibiting peripheral arthritis. However, a case series has reported flares of arthritis, sacro‐ileitis, or both with vedolizumab [215].

Unlike biologics, which are typically administered intravenously or subcutaneously, synthetic small‐molecule drugs—such as JAK inhibitors and S1P receptor modulators—are gaining prominence as effective oral treatments for IBD. JAK, including JAK1, JAK2, JAK3, and TYK2, are enzymes linked to the intracellular domains of cytokine receptors [216]. JAK inhibition, therefore, has the potential to affect multiple proinflammatory cytokine‐dependent pathways. Tofacitinib is a pan‐JAK inhibitor, which, acting either on JAK1 and JAK3 enzymes, blocks signals from IL‐12, IL‐23, and other cytokines by inhibiting downstream signaling pathways. It has received US FDA and EMA approval based on the positive results from clinical trials (OCTAVE studies) demonstrating its efficacy in UC [217]. In contrast, two Phase IIb randomized placebo‐controlled clinical trials showed no superiority of tofacitinib over placebo for CD. This pattern—clear UC efficacy paired with the absence of CD benefit—is consistent across the JAK‐inhibitor class, suggesting that the JAK/STAT signaling pathways differ qualitatively between the two diseases [218].

However, safety concerns related to the risk of thrombosis and pulmonary embolism, associated with pan‐JAK inhibitors like tofacitinib [219], are propelling the development of more selective JAK1 inhibitors. Upadacitinib and filgotinib, both selective JAK1 inhibitors, have been approved by the US FDA and EMA for the management of patients with moderate‐to severe UC, based on the results of Phase III clinical trials. U‐ACHIEVE and U‐ACCOMPLISH studies [220] showed that, in both, induction and maintenance phases, a significantly greater proportion of patients receiving upadacitinib achieved clinical remission compared with placebo (*p* < 0.0001) [220]. The SELECTION study [221] demonstrated that filgotinib was more effective than placebo in inducing remission at Week 10 among patients without prior exposure (26.1 vs. 15.3%, *p* = 0.02) and those with prior exposure (11.5 vs. 4.2%, *p* = 0.01) to a TNF antagonist or vedolizumab. Additionally, filgotinib demonstrated superior maintenance of clinical remission compared with placebo (37.2 vs. 11.2%, *p* < 0.001) [221].

At present, upadacitinib is the only JAK inhibitor approved for the treatment of CD, supported by the positive results from two Phase III induction trials (U‐EXCEL and U‐EXCEED) as well as and the Phase III maintenance trial (U‐ENDURE) [222]. More recently, the Phase III‐DIVERSITY study [223] showed that filgotinib was not effective as induction therapy in CD patients; however, at a dose of 200 mg once daily, it showed efficacy in achieving clinical remission and endoscopic response during the maintenance phase. Treatment with JAK inhibitors may be associated with decreases in absolute lymphocyte and neutrophil counts, reductions in hemoglobin levels, and alterations in liver enzymes (cytolysis) [224].

S1P receptor modulators work by binding to S1P receptors on endothelial cells and lymphocytes, inducing receptor internalization and degradation. This process blocks lymphocyte egress from lymph nodes into the bloodstream, resulting in reduced circulating lymphocytes and subsequently decreased inflammation and tissue damage. Among these modulators, ozanimod and etrasimod have received US FDA and EMA approval for effectively inducing and maintaining remission in UC. The Phase III TRUE NORTH study [225] showed that patients treated with ozanimod achieved significantly higher rates of clinical remission during both the induction phase (Week 10) and maintenance phase (Week 52) compared with placebo (*p* < 0.001). Furthermore, the OLE study [226] revealed sustained benefits, with 91.4% of patients maintaining clinical response, 69.1% maintaining clinical remission, and 67.9% remaining corticosteroid‐free at Week 94 of the OLE (146 weeks total treatment).

The ELEVATE study [227] demonstrated etrasimod effectiveness in ameliorating clinical remission at induction (Week 12: 27 vs. 7%) and at maintenance (Week 52: 32 vs. 7%) in UC patients compared with patients in the placebo group. Due to the risk of adverse effects, regular monitoring is required, including electrocardiograms, heart rate, blood pressure (hypertension was more frequently reported in clinical trials), blood count, and liver function tests.

Despite the expansion of the IBD therapeutic armamentarium, it remains difficult to extrapolate data from clinical trials to guide the choice and positioning of the most appropriate treatment for individual IBD patients. This stems from several factors, including a shift in clinical‐trial endpoints. Over the past two decades, research methodology has gradually evolved from focusing on symptom control to addressing the underlying inflammatory process, with the ultimate goal of preventing disease progression and complications. In this regard, STRIDE‐II guidelines [228] promote treat‐to‐target strategy with short‐term (symptom relief, biomarker normalization) and long‐term goals (mucosal healing, remission, no disability). While efficacy in IBD trials traditionally emphasized symptomatic improvement, more recent trials incorporate objective measures of disease activity, such as histological remission [227, 229], histological‐endoscopic remission [227, 230, 231], histological and endoscopic mucosal healing [227, 232] in UC patients and histological remission/healing [233, 234], and transmural remission/healing [234, 235, 236] in CD patients. However, several factors limit the applicability of the treat‐to‐target strategy in real‐world practice; these include the absence of a validated definition of mucosal healing in both CD and UC, the limited feasibility of repeated endoscopy for tight disease and therapeutic drug monitoring, and the lack of reliable biomarkers as alternatives to endoscopy.

Mapping current biologics and small molecules onto the modern STRIDE‐II endpoint hierarchy clarifies their respective strengths and limitations (Table 2).

**TABLE 2:** Evidence mapping of advanced therapies across the STRIDE‐II endpoint hierarchy in UC and CD.

		Tier 1: Clinical remission		Tier 2: Endoscopic remission/response		Tier 3: Histologic remission	Tier 4: Transmural healing
Drug class	Agent(s)	Ind.	Maint.	Ind.	Maint.	Remission	Healing
**Ulcerative colitis (UC)**							
Anti‐TNFα	Infliximab, adalimumab, golimumab	Robust	Robust	Robust	Robust	Partial	No data
Anti‐integrin (α4β7)	Vedolizumab	Robust	Robust	Robust	Robust	Partial	No data
Anti‐IL‐12/23 (p40)	Ustekinumab	Robust	Robust	Robust	Robust	No data	No data
Anti‐IL‐23 (p19)	Mirikizumab, risankizumab	Robust	Robust	Robust	Robust	Robust	No data
JAK inhibitors	Upadacitinib, tofacitinib, filgotinib	Robust	Robust	Robust	Robust	Partial	No data
S1P receptor modulators	Ozanimod, etrasimod	Robust	Robust	Robust	Robust	Partial	No data
**Crohn's disease (CD)**							
Anti‐TNFα	Infliximab, adalimumab, certolizumab	Robust	Robust	Robust	Robust	Partial	Robust
Anti‐integrin (α4β7)	Vedolizumab	Partial	Robust	Limited	Partial	No data	No data
Anti‐IL‐12/23 (p40)	Ustekinumab	Robust	Robust	Robust	Robust	No data	Limited
Anti‐IL‐23 (p19)	Risankizumab, mirikizumab	Robust	Robust	Robust	Robust	No data	Limited
JAK inhibitors (selective JAK1)	Upadacitinib	Robust	Robust	Robust	Robust	Partial	No data
S1P receptor modulators	Ozanimod, etrasimod	−	−	−	−	−	−

The relative position of each drug class along the endpoint hierarchy (clinical → endoscopic → transmural/histologic). Each cell reflects the depth of published evidence for that drug class at the given STRIDE‐II endpoint tier, based on pivotal Phase III randomized controlled trials and available long‐term extension data. Robust evidence = endpoint met as primary or key secondary outcome in ≥1 registration trial. Partial/emerging = positive signal in exploratory analyses, limited‐sample real‐world data, or a single smaller trial. Limited = posthoc or subgroup data only. No data = endpoint not prespecified or not reported. Transmural healing data are accruing for several classes (ongoing FORTIFY‐OLE, U‐ACTIVATE, LUCENT‐3 studies). STRIDE‐II, Selecting Therapeutic Targets in Inflammatory Bowel Disease version II [228].

Abbreviations: JAK, Janus kinase; S1P, sphingosine‐1‐phosphate; IL, interleukin; TNF, tumor necrosis factor; UC, ulcerative colitis; CD, Crohn's disease.

Anti‐TNF agents (infliximab, adalimumab) remain best positioned for rapid induction of clinical and endoscopic remission and have the most robust evidence for transmural healing in CD when combined with immunomodulator therapy, as shown by the recent PROFILE trial [127]. Anti‐IL‐12/23 (ustekinumab) and selective anti‐IL‐23p19 antibodies (risankizumab, mirikizumab) achieve the highest histological‐endoscopic remission rates in UC and produce durable transmural improvement in CD (UNIFI, LUCENT‐1/2, ADVANCE/MOTIVATE programs). Vedolizumab consistently outperforms adalimumab for endoscopic remission in UC in the head‐to‐head VARSITY trial but provides only modest deep‐healing efficacy in CD, in line with its gut‐selective α4β7‐trafficking mechanism. JAK inhibitors (tofacitinib, upadacitinib, filgotinib) deliver the fastest symptomatic and clinical response, but transmural healing data are currently sparse, and class‐wide safety considerations (cardiovascular events, herpes zoster reactivation, lipid changes) [224] constrain their use as first‐line agents in elderly or cardiovascular‐risk patients. S1P modulators (ozanimod, etrasimod) achieve clinical and endoscopic remission in UC with a more favorable cardiovascular profile than JAK inhibitors, but no head‐to‐head data are yet available to position them definitively. Combination strategies (e.g., the VEGA proof‐of‐concept trial of guselkumab plus golimumab [237], raise the question of whether dual targeting of complementary pathways can lift deep‐remission rates above the ceiling observed with any single agent—a question that will likely shape the next phase of IBD therapeutic development.

Furthermore, the marked heterogeneity in IBD trials given not only to different endpoint definitions, but also to different clinical trial design, patient populations, previous treatment exposure, data acquisition, and analytical approaches, makes it difficult to compare clinical trial results of different therapeutic agents in IBD.

These limitations highlight the need for more robust comparative studies to guide optimal therapy selection. Randomized head‐to‐head trials are therefore essential to optimize therapeutic strategies in IBD. Despite the availability of multiple mechanisms of action, comparative effectiveness data remain sparse, with only a few head‐to‐head trials offering direct guidance on sequencing therapies.

This points to the need to integrate data from the few available head‐to‐head clinical trials [238] (e.g., SEAVUE, VIVID‐1 for CD and VARSITY and for UC) with network meta‐analysis and real‐world comparative effectiveness and safety studies (which compare the effectiveness and safety of treatments in routine clinical practice, in diverse patient populations) as general approach to positioning therapies.

### 3.2 Emerging Therapies

Most existing therapies focus on suppressing the immune response, but reducing immune suppression could offer a novel direction, since immune function is essential for health. Emerging mucosal‐repair approach such as targeting gene‐expression, using MSCs, and transplanting intestinal microbiota (Table 3), aims to enhance mucosal healing and complement current treatments to improve overall efficacy (Table 4).

**TABLE 3:** Preclinical studies of emerging nonimmune‐targeted therapies for inflammatory bowel disease.

IBD model	Animal species	Modeling duration	Treatment	Route	Times of treatment	Times of observation	Dose	Explored mechanism of action	Main results	References
DSS‐induced acute and chronic colitis	C57BL/6 mice	Acute model = 8 days DSS + 3 days water Chronic model = 63 days DSS	ABX464 (obefazimod)	OS	Once a day (from the start to the end of DSS challenge) either in acute and chronic models OR once a day for the first 20 days in chronic model	Day 11 (acute model) Day 63 (chronic model)	50 mg/kg in methylcellulose	Anti‐inflammatory action	Treatment reduced the colonic production of the inflammatory cytokines IL‐6 and TNFα as well as that of the chemoattractant MCP‐1. ABX464 to induce the expression of IL‐22.	[242]
DSS‐induced acute colitis	C57BL/6 mice	7 days DSS + 3–5 days water	ABX464 (obefazimod)	OS	Once a day for the duration of the study	Day 12	40 mg/kg in methylcellulose	Anti‐inflammatory action	Treatment reduced disease severity in DSS‐induced colitis. Treatment significantly reversed the increases in several proinflammatory cytokines induced by DSS. Furthermore, treatment reversed the DSS‐induced upregulation of IL17a‐secreting cells and of IFNγ‐secreting CD4+ cells in mesenteric lymph nodes.	[243]
Colitis model induced by transfer of naive T cells into C57BL/6J Rag1‐deficient mice	Germ free C57BL/6J C57BL/6J Rag1‐deficient mice	1 × 10^6^ CD45RB^hi^T cells in 200 µL of sterile PBS by IP in C57BL/6J Rag1‐deficient mice	Human fecal transplantation from human healthy and IBD patients	Intragastric	FMT only once after weaning (28–42 days old) T‐cell transfer at least 28 days after colonization	7 days after T cell transfer	200–300 µL of a fecal slurry	Effects on immune landscape in the gut	(1) Transfer of IBD microbiotas into germ‐free mice increased numbers of intestinal Th17 cells and Th2 cells and decreased numbers of RORγt+ Treg cells. (2) Transplantation of fecal samples from IBD patients exacerbated disease in a model of colitis induced upon transfer of naive T cells into Rag1−/− mice.	[251]
No disease model	Germ‐free C57Bl/6 mice	No disease model	Stool from CD mothers or their respective 3‐month‐old baby	Intragastric	Once	5 weeks after fecal transplantation	200–300 µL of human fecal slurry	Colonic immune system alterations	Germ‐free mice inoculated with the stool of mothers with CD and their 3‐month‐old babies have significant abnormalities in the adaptive immune cells compared with mice inoculated with stool from non‐CD controls. Germ‐free mice receiving IBD‐associated stools showed reduced levels of colonic TREG and IgA+ B cell subsets.	[252]
TNBS‐induced colitis	SPF mice		Transplant with 17 strains of bacteria within clusters IV, XIVa, and XVIII of *Clostridia* isolated from human microbiota	Intragastric	Not reported	Day 4 after TNBS	Not reported	Tissue preservation	Colitic mice receiving 17‐mix bacteria strains showed less severe colon shortening and milder histological disease features, accompanied by lower mortality. Increased levels of Foxp3 and Tgfb1 mRNA and a tendency toward a reduction of inflammatory cytokine transcripts were observed.	[253]
DSS‐induced acute colitis	C57BL/10 mice	DSS for 7 days	Feces from healthy C57BL/10 donor mice	Intragastric	Once a Day or 7 d after DSS challenge	Day 14	0.1 mL/10 g BW /mouse	Gut microbiota composition and gut immunity	FMT significantly reduced weight loss, colon length shortening and inflammation in colitic mice. FMT upregulated the relative abundance of Lactobacillus and downregulated the relative abundance of *Clostridium sensu stricto 1* and *Turicibacter* in gut microbiota. FMT reduced colonic levels of CD4+ and CD8+ lymphocytes.	[254]
DSS‐induced acute colitis	C57BL/6 and CXCR6‐EGFP/+ mice	DSS for 7 days	Fecal microbiota transplantation from normobiotic donor mice	Intragastric	For 3 consecutive days starting 1 day after the end of DSS administration	Day 11 and Day 15	10 mg/mouse	Gut microbiota composition and gut immunity	FMT ameliorated DSS‐induced experimental colitis (in terms of weight loss, colon length shortening and histological score). In FMT‐treated colitic mice, a restoration toward normo‐biosis was observed (less abundant *Firmicutes*, *Clostridiaceae*, and *Clostridiales* families and increased levels of probiotic commensals). FMT‐treated mice showed: reduced levels of colonic CD4+ cells, macrophages, dendritic cells, and neutrophils, while increased colonic IL‐10 levels together with higher frequence of IL‐10‐producing APCs, CD4+ T cells, and iNKT cells.	[255]
DSS‐induced chronic colitis	C57BL/6 and CXCR6‐EGFP/+ mice	3 cycles of 7 days of DSS followed by 14 days of water recovery.	Fecal microbiota transplantation from normobiotic donor mice	Intragastric	3 cycles of 3 consecutive doses each after 5 days after the beginning of each DSS cycle	Day 63	10 mg/mouse	Gut microbiota composition and gut immunity	FMT did not meliorate DSS‐induced chronic colitis (in terms of weight loss and histological score); only a mild increase in colonic length was observed. At sacrifice, in FMT‐treated mice, no variations in levels of Invariant natural killer T (iNKT) and conventional CD4+ and CD8+ T cells were observed. However, FMT reduces immune cell proinflammatory cytokine profile. FMT restored normobiosis both in terms of microbial composition and metabolic functions.	[256]
DSS‐induced acute colitis	BALB/c mice	DSS for 7 days	Human fecal FMT from healthy donors	Intragastric	Once a day for 8 consecutive days starting from the first day of DSS administration	Day 9	0.1 mL/10 g/mouse	Immunoglobulin A‐targeted microbiota	FMT significantly ameliorated clinical symptoms (DAI, body weight) and histological score in colitis mice. After FMT, both IgA/G B cell and the percentage of IgA/G‐targeted bacteria are reduced to normal levels in DSS mice.	[257]
DSS‐induced acute colitis	C57BL/6	DSS for 7 days	Fecal microbiota transplantation from normobiotic donor mice	Intracolonic injection	Once a day for the next 7 days after the end of DSS administration	Day 14	100 g/50 kg per mouse	Gut microbiota composition and gut immunity	FMT therapy significantly relieved the symptoms of DSS‐induced colitis in mice. FMT alleviated the microbiota alterations caused by DSS. FMT treatment restored the expression of AhR in Treg cells associated with increased colonic levels of IL‐10 and TGF‐β.	[258]
Colonization with microbiotas from human donors with IBD	Germ‐free C57Bl/6 mice	3 weeks	Transplant with human healthy donor microbiota	Intragastric	3 week later from colonization with IBD‐derived microbiota	6 weeks (3 weeks after FMT)	Not reported	Impact of FMT on the host intestine tissue resident immune populations	FMT therapy in mice colonized with IBD microbiotas can lead to an increase in the proportion of RORγt+ Treg cells and suppressed the proportion of gut Th17 cells. FMT modulated an IBD‐associated Th17‐inducing strain microbiota (*E. coli* strain A6).	[259]
DSS‐induced acute colitis	C57BL/6 mice	DSS for 7 days + water for 3 days	Syngeneic murine BM‐MSCs	IP	One treatment (Day 7)	Day 10	1 × 10^6^ MSCs/mouse	MSCs did not exhibit the expected immunomodulatory functions.	MSC treatment did not ameliorate survival rate and DAI scores. In addition, MSCs did not change the % of cells expressing interferon (IFN)‐γ, interleukin (IL)‐4, IL‐6, IL‐10, and IL‐17, and Treg cells either in spleen and in mesenteric lymph nodes. Nor the treatment changed the levels of cytokines expressed in colonic tissues. NB: the number of samples analyzed were *n* = 2.	[275]
TNBS‐induced chronic colitis	Balb/c mice	Increasing TNBS concentrations every 7 days for a total of 7 weeks	Syngeneic murine BM‐MSCs	IP	Prophylactic treatment = 24 h before each TNBS infusion Therapeutic treatment = every day for 7 days after 7 weeks of TNBS infusions	Day 59 (10 days after the last TNBS infusion)	1 × 10^6^ MSCs/dose	Antifibrogenic effect	In this model, prophylactic MSC treatment inhibited the accumulation of fibrotic tissue and the therapeutic treatment reversed the established fibrosis. Intestinal fibrosis was evaluated as mRNA colon expression of fibronectin and collagens. Reductions in mRNA expression of inflammatory cytokines and of TGF‐beta were also observed. Furthermore, decreased levels of TGF‐beta, p‐Smad2, and p‐Smad3 were also found in colon tissues.	[276]
DSS‐induced acute colitis DSS‐induced relapsing colitis	C57BL/6 mice	Acute model = DSS for 6 days + water for 9 days Relapsing model = 2× cycles (DSS for 6 days + water for 9 days)	Allogenic (from BALB/c mice) or syngeneic BM‐MSC	IP, SC, or IV	Acute model = 2 treatments (Days 2, 4) Relapsing model = 2 × 2 treatments (1° cycle: Days 2, 4; 2° cycle: Days 18, 20)	Acute model = Day 15 Relapsing model = Day 30	1 × 10^6^ MSCs/dose (IV) 10 × 10^9^ MSCs/dose (IP, SC)	No mechanism of action was explored	The administrations of syngeneic MSCs by IP and SC were more effective in ameliorating mice colitis (lower BW loss, higher DAI, and histological scores) than IV treatment (NB: lower dose of MSCs when IV injected). Both, allogeneic and syngeneic MSCs, IP injected during the first cycle of DSS‐challenge, ameliorated mice colitis in equivalent manner. When instead MSCs were administered during the second cycle of DSS‐challenge (relapsing model), allogenic MSCs did not ameliorated mice colitis, in contrast syngeneic MSCs displayed significant clinical improvement with improved body weight, DAI, and histological scores.	[277]
DSS‐induced acute colitis	C57BL/6 mice	DSS for 6 days + water for 4 days	Syngeneic murine BM‐MSCs primed (cytokine cocktail) or not primed or EV from primed or not primed MSC	IP	Two treatments MSCs (Days 4, 8) Three treatments EVs (Days 4, 6, 8)	Day 10	4 × 10^6^ MSCs/dose 1 × 10^9^ EVs/dose	Macrophage and T cell polarization; protective/regenerative effect of intestinal mucosa	The administration of MSCs, primed or not, worsened murine colitis evidenced by weight loss and colon length reduction with a trend to increased DAI and increased histological score. In contrast, EV treatments improved clinical and histological signs of colitis. EVs from primed MSCs were more effective than EVs from not primed MSCs. Both MSCs reduced CD45+ cell infiltration, but not promote the polarization of macrophages toward M2 nor polarization of T cells toward Treg. In contrast, EV treatments did not change CD45+ cell infiltrations, but, mainly, EVs from primed MSCs promoted macrophage and T cell polarization toward regulatory phenotypes (M2 and Treg). In colon mucosa, unprimed MSCs and EVs from primed MSCs induced protein expression of MUC5 suggesting improvement of epithelial function.	[278]
TNBS‐induced acute colitis	Balb/c mice	Presensitization for 7 days, acute model = intrarectal TNBS (Day 0) Chronic model = intrarectal TNBS (Days 0, 7, 14, 21)	Human BM‐MSCs	IV and IP	One treatment (24 h before TNBS enema Day 0)	Acute model = Day 2 Chronic model = Day 23	1 × 10^6^ MSCs	Increment of IL‐10‐producing B cells through THBS1‐mediated TGF‐β activation	MSCs delivered by IP route, with respect to those delivered by IV, more efficiently counteracted development of acute colitis. IP‐MSCs reduced DAI, histological score, the colonic levels of proinflammatory cytokines (TNF‐α, IFN‐γ) and neutrophil infiltration (MPO activity). A single IP injection of MSCs was able to also counteract development of chronic colitis, indeed treated mice showed lower DAI, reduced histopathological score and reduced colonic levels of proinflammatory cytokines. In the acute model, the authors showed that MSCs increased the intraperitoneal levels of IL‐10‐producing B cells and when B cells were depleted in the peritoneal cavity, MSC‐mediated colitis alleviation was abolished. Moreover, the authors showed that MSCs knockout of THBS1 had little effect against the severity of colitis and lost their ability to induce IL‐10 expressing Breg. Furthermore, MSCs lost their benefits and their ability to promote B cell polarization, when TGF‐β pathway was inhibited by blocking TGF‐βR. These data suggested that MSC‐derived THBS1 could boost IL‐10‐producing B cells through THBS1‐mediated TGF‐β activation.	[279]
Chronic and spontaneous murine model	SAMP‐1/YitFc mice	From established disease (>24 weeks old mice, Day 0) to Day 28	Human bone marrow‐derived MSCs and dexamethasone (positive control)	IP	hMSCs: one treatment (Day 0) early dexamethasone (7 daily doses before Day 0) late dexamethasone (7 daily doses before Day 28)	Day 9 and Day 28	5.0 × 10^6^ MSCs	Macrophage polarization via efferocytosis	Live hMSCs are present until Day 9 from IP injection in SAMP mice, while no viable hMSCs were detected on Day 28. At Day 9 after treatment, hMSCs reduced the percent of abnormal mucosa, reduced CD4 T cell levels and gene expression of proinflammatory cytokines (TNF‐α, IL‐4, IL‐21) in mesenteric lymph node. Similar results were observed after early treatment with DES. At Day 28 after treatment, hMSCs still reduced percent of abnormal mucosa, reduced the histopathological index and decreased cytokines levels in mesenteric lymph nodes. DES treatment performed early did not ameliorate the disease parameters, amelioration was observed only when DES was delivered just prior sacrifice time (Day 28). Thus, hMSCs showed long‐lasting effects in chronic colitis murine model. Moreover, in SAMP peritoneal fluid after intraperitoneal injection of hMSCs, higher levels of anti‐inflammatory (arginase‐I+) and phagocyting (Gas7+) macrophages were observed suggesting that macrophage efferocytosis of apoptotic hMSCs can reprogramme macrophages toward anti‐inflammatory phenotype.	[280]
TNBS‐induced acute colitis	BALB/c mice	Mono‐intrarectal enema of TNBS	Human umbilical cord‐MSCs	IP	One dose, 2 h after TNBS enema	Days 3, 6	Three different doses: 5 × 10^5^, 1 × 10^6^ and 2 × 10^6^ hUC‐MSCs	Modulation of Th1/Th2/Th17 cells and of T regulatory 1 (Tr1) cells	Dose–response effect in terms of amelioration of DAI scores and histological scores and maintenance of colon length. Lower relative abundance in Th1 and Th17 cells and higher % of T regulatory 1 cells (Tr1) in spleen and MLN of treated mice. The maximal effect was observed with the higher MSC dose used. The effect on Th1 and Th17 was observed only at Day 3 and not at Day 6 (after TNBS). Tr1 proportions were instead higher at Day 6 with respect to Day 3 after TNBS. hUC‐MSCs with IDO‐knockdown lost both, the clinical benefits and the effect on Tr1.	[281]
DSS‐induced acute colitis	C57Bl/6 mice	7 days	Human umbilical‐cord‐derived MSCs primed or not with IFN‐γ and TNF‐α	IV	One treatment (on Day 3)	Day 7	1 × 10^6^ primed or not primed hUC‐MSCs	Immunosuppression by upregulating MSC expression of programmed death‐ligand 1 (PD‐L1)	hUC‐MSCs ameliorated DSS‐induced colitis in terms of lower histopathological score; reduced infiltration of CD4+ T cells, CD11b+ macrophages, and neutrophils (lower MPO activity) and increased mRNA expression of Foxp3 (marker of Treg) in colon tissue. Moreover, treated mice showed decreased levels of inflammatory cytokines such as TNF‐α, IFN‐γ, and IL‐6 in colon tissue. Primed MSCs were more effective than not primed. The cotreatment with PD‐L1 antibody eliminated the benefits exerted by primed MSCs on colitic mice, suggesting the involvement of PD‐L1 in MSC activity.	[282]
DSS‐induced acute colitis	BALB/c mice	7 days	Exosomes (Exos) from human umbilical cord‐MSCs and exosomes human placenta‐MSCs	IV	Daily for 7 days at the end of Day 7‐DSS treatment	Days 7, 14	50 µg (total proteins) exosomes/dose	Modulation of Treg in peripheral blood and modulation of gut microbiota	Exos from UC‐MSCs and from placenta‐MSCs, both alleviated DSS‐induced mouse colitis in terms of reduction in histopathological score; in plasma levels of cytokines (IL‐6, IFN‐γ, IL‐17A, and IL‐10) and in the gene expression of the same cytokines in colon tissues. IL‐23 and IL‐4 gene expressions were also increased in the colon of treated mice. It is to be underlined, that in this study, the infusion of UC‐Exos or P‐Exos decreased the % of CD4 + Foxp3 + cells and CD8 + Foxp3 + cells in peripheral blood of treated mice. Infusion of hUC‐Exos and hFP‐Exos reduced the abundance of proinflammatory bacterial, such as *Verrucomicrobia*, *Akkermansia*, *A. muciniphila*, and *Escherichia coli*, and promote the reestablishment of the intestinal microbiota homeostasis.	[283]
DSS‐induced acute colitis	C57Bl/6 mice	7 days	Exosomes derived from murine adipose‐MSC	IP	Three times (on Days 2, 4, and 6)	Day 7	100 µg (total proteins) exosome/dose	Anti‐inflammatory action via suppression of inflammatory cytokine secretion and increased relative Treg levels.	Exo treatment decreased the disease activity index, the histological score and MPO activity (indicator of neutrophil infiltration in the colon tissue). Exo treatment also decreased the secretion of inflammatory cytokines (TNF‐α, IFN‐γ, IL‐17, IL‐12) and increased the secretion of TGF‐β, IL‐10, and IL‐4, from activated (with PHA) mononuclear cells isolated from spleen and MLN. Moreover, mice treated with Exos showed higher relative amount of Treg in MLN and spleen.	[284]
TNBS‐induced colitis	BALB/c mice	Mono‐intrarectal enema of TNBS	Human adipose MSCs	IP	Once (24 h after TNBS enema)	Days 3, 7, 14, or 60	1 × 10^6^ cells/mouse	Treg recruitment and Th1/Th17 inhibition	During 14 days after TNBS, mice treated with MSCs gained weight, showed improvement in the clinical disease score and exhibited reduced mortality rate. They also transitorily reduced inflammatory colon infiltration and increased % of goblet cells. On Day 60, the inflammatory infiltrate was similar in treated and untreated animals. The ability of MSCs to maintain goblet cells was maintained. No effect on Treg levels were observed, while reduced levels of Th17 T cells in mesenteric lymph nodes were found.	[285]
TNBS‐induced perianal fistula model	Sprague–Dawley rats	Intrarectal TNBS administration twice a week for 1 week + surgical creation of fistulas (Day 0) + TNBS administration through the fistula tracts twice a week for 48 days	Fragmented nanofiber‐hydrogel composite loaded with syngeneic adipose‐MSCs + fragmented nanofiber‐hydrogel composite loaded without adipose‐MSCs	Intrafistula injection	One treatment (Day 28 after fistula creation)	Day 48 (after 20 days from treatment)	1 × 10^5^ MSCs/mouse	Anti‐inflammatory effects	Treatment with gel loaded with MSCs showed he higher degree of fistula closure, the lower inflammatory infiltrate and the higher neo‐vessel formation. In addition, this treatment reduced mRNA levels of proinflammatory cytokines such as TNF‐α, IL‐6, IL‐1α, IFN‐γ, IL‐1β, IL‐23α in fistula tissues. Furthermore, the treatment reduced the infiltration of T cell, B cell, NK cell, and myeloid cells and reduced protein levels of IL‐6, TNF‐α, IL‐17, CC3, and IL‐1Ra in fistula tissues.	[286]
TNBS‐induced acute colitis	Sprague–Dawley rats	Rectal administration of TNBS (Day 0)	Human amnion‐derived MSCs (hAMSC) or CM from hAMSCs (in gel of carboxymethyl cellulose)	IV (MSCs) or intrarectal administration (CM)	One treatment (MSCs: 3 h after TNBS) Three treatments (CM: 3 h, Day 1, Day 2 after TNBS)	Day 7	1 × 10^6^ MSCs/dose 400 µL of CM gel/dose	Anti‐inflammatory effects	Administration of MSCs in TNBS‐challenged mice ameliorated endoscopic score, reduced histological score and levels of neutrophils (MPO+ cells), of macrophages (CD68+ cells) and of T lymphocytes (CD3+ cells). MSCs transplantation tended to decrease the mRNA expression of TNF‐α, CXCL1, CCL2, and IL‐6 in colon tissues. CM‐gel showed effects equivalent to MSCs administration.	[287]
DSS‐induced acute colitis	C57BL/6 mice	DSS for 9 days + water for 7 days	CM from human uterine cervical MSCs (loaded on a mucoadhesive and thermosensitive hydrogel) or control medium loaded on hydrogel or mesalazine (5‐aminosalicylic acid) foam	Rectal administration	Twice daily (from Day 9 to Day 16)	Day 16	75 µL of CM‐MSC or CM‐control loaded on hydrogel	Anti‐inflammatory action	Hydrogel loaded with CM‐MSCs reduced histological alterations in mice colon, mainly in distal location (with respect to cecum); reduced inflammation score in all colon sections (more effectively than mesalazine); reduced mRNA levels of proinflammatory cytokines (TNF‐α, IFN‐γ, and IL‐6) in colon tissues.	[288]
TNBS‐induced acute colitis	BALB/C mice	No presensitization, TNBS enema (Day 0)	MSCs derived from human iPSC (iPSC‐MSCs) iPSC‐MSCs with TSG6 knockdown	IP	One treatment 24 h after enema	Day 5 after enema	2 × 10^6^ cells	Mucosal healing via tumor necrosis factor‐α‐stimulated gene 6 (TSG‐6)	The administration of MSC‐iPSCs ameliorated mice colitis in terms of reduced loss of body weight, reduced ulcer size and histological score. iPSC‐MSCs secreted TSG6 and they lost their therapeutic effects after TSG‐6 knockdown. Moreover, treatment with MSC‐iPSCs increased the number of CD44+ epithelial cells and of proliferating epithelial cells (Ki67+ cells); increased also the numbers of both Lgr5‐positive (Lgr5+) cells and Smoc2‐positive (Smoc2+) cells (markers of cycling crypt base columnar cells). Even in this case, treatment with iPSC‐MSCs knockdowned for TSG6, did not increase the proliferation and activation of CBC stem cells. Treatment with recombinant human TSG6 showed clinical and mucosal healing benefits similar to iPSC‐MSCs. Since the coadministration of PEP‐1 blocking of CD44 from interactions with HA abrogated the therapeutic effects of iPSC‐MSCs or rhTSG‐6, the authors suggested that TSG‐6 secreted by iPSC‐MSCs promoted the proliferation of epithelial cells in colonic crypts through interactions between CD44 and hyaluronic acid.	[289]
DSS‐induced acute colitis	CD‐1 mice	DSS for 19 days	iPSC‐derived MSCs and adipose‐derived MSCs	IV	Three treatments (Days 10, 13, and 16)	Day 19	1 × 10^6^ MSCs/dose	Partial restoration of normal colonic microbiome	iPSC‐MSCs were equivalent to adipose‐MSCs to improve intestinal colitis alterations. Treatments improved both clinical and histological inflammatory scores (with reduced colonic levels of numbers of macrophages and monocytes, increased levels of Treg, no effects on T effector and B cell levels). Increased levels of proliferating (Ki67+ cells) epithelial cells, of intestinal stem cells (Lgr5+ cells) and of endothelial cells (CD31+ cells) were found in treated mice. In addition, MSC treatments helped the restoration of the normal colonic flora; they indeed reduced colonic levels of *Proteobacteria* and enhanced the colonic microbial diversity, overall iPSC‐MSCs.	[290]
DSS‐induced acute colitis	C57BL/6 mice and BALB/c mice	DSS for 10 days	Murine syngeneic BM‐MSCs and murine BM‐MSC encapsulated in barium alginate and murine adipose‐MSCs (syngeneic and allogeneic)	IV or subcutaneous injection	One treatment (Day 5)	Day 10	3 × 10^6^ MSCs	Anti‐inflammatory action via increased IL‐10 expressing macrophages and Treg levels mediated by TSG‐6.	Failure of BM‐MSCs to ameliorate DSS‐induced colitis after intravenous injection. Amelioration of disease parameters (body weight loss, DAI, histological score) after subcutaneous injection of BM‐MSC and also adipose‐MSC (either syngeneic and allogeneic). The effects were maintained even after MSC encapsulation. TSG‐6 serum levels were significantly higher in MSC‐treated mice compared with controls at 48 and 72 h after injection and MSCs deficient in TSG‐6 lost their therapeutic effects, confirming TSG‐6 as the critical factor through which MSCs exert their anti‐inflammatory effects. MSCs and exogenously delivered recombinant murine TSG‐6 promoted the expansion of colonic macrophages with a regulatory phenotype (IL‐10 expressing macrophages) and enhanced the percentage of Foxp3CD45þ cells (Tregs) in the mucosa and in the mesenteric lymph node.	[295]
DSS‐induced acute colitis	C57BL/6J mice	7 days DSS + 3 days water	Human adipose MSCs and adipose‐MSCs transfected with TSG‐6 siRNA	IP	One treatment (on Day 1)	Day 10	2 × 10^6^ MSCs	Macrophage polarization	Treatment with adipose‐MSC ameliorated DSS‐induced colitis in terms of lower DAI, lower histologic scores, more preserved colon length. In addition, the administration of adipose‐MSCs decreased the gene expression of TNF‐α, IL‐1β, IFN‐γ, and IL‐17, and increased the expression of IL‐10 in colonic tissue. Furthermore, treatment increased the mRNA expression of M2 macrophage markers (CD206, Arg1, Fizz1, and Ym1) in colon tissue, associated with a reduced number of CD11b and CD206‐positive cells within the inflammatory colonic infiltrates. In contrast, the administration of adipose‐MSCs with TSG‐6 knockdown neither improved the disease parameters nor promote macrophage polarization toward M2 phenotype. These data suggested that TSG‐6 may play a role in M2 macrophage polarization in DSS‐induced colitis mice.	[296]
DSS‐induced acute colitis	NOD/SCID mice	5 days DSS + 2 days water	Conditioned medium (CM) derived from amniotic fluid‐MSCs	IP	One treatment (at Day 5)	Day 7	200 µL	Anti‐inflammatory action	CM administration at the onset of the DSS‐colitis, improved the clinical and histopathologic severity of colitis; downregulated the mRNA expression of proinflammatory cytokines such as TNF‐α, and IL‐1β and increased mRNA expression of the anti‐inflammatory cytokine IL‐10 in colon tissues. TNF‐α and MMP2 protein levels were reduced while TGFβ levels were increased in colon tissues.	[297]
DSS‐induced acute colitis	C57BL/6 mice	DSS for 7 days	CM derived from hydrogen peroxide (H_2_O_2_)—primed or not primed murine BM‐MSCs	By enema	Three treatments (Days 2, 4, and 6)	Day 8	200 µL CM	Antioxidant activity by modulation of Nrf2/Keap1/ARE signaling pathway	Priming of MSCs with H_2_O_2_ increased MSC expression of Nrf2 and its downstream proteins heme oxygenase 1 and quinone oxidoreductase 1, possibly by activating the intracellular Nrf2/Keap1/ARE signaling pathway. The anticolitic effects of CM from primed MSCs were higher than those of CM from not primed MSCs. The pretreatment with an Nrf2 inhibitor (ML385), abolished the benefits (reduced DAI and histological score and reduced inflammatory infiltration) of CM from primed MSCs. In addition, the pretreatment with ML385 abolished the improvement of intestinal mucosal barrier (ZO‐1 expression) and of oxidative stress (ROS and MDA levels and antioxidant enzymes).	[300]
DSS‐induced acute colitis	C57BL/6 mice	DSS for 7 days	Murine BM‐MSCs or CM derived from murine BM‐MSCs or ML385‐treated BM‐MSC	By enema or IP (ML385‐MSCs)	Two treatments (Days 4 and 6)	Day 8	1 × 10^6^ MSCs/dose or 200 µL CM	Antioxidant activity by modulation of Nrf2/Keap1/ARE signaling pathway	The comparison between the efficacy of BMSC and CM delivered by enemas in colitis, indicated that cells are more effective than CM in improving clinical and inflammatory parameters (DAI, histological score, CD68 and neutrophil infiltration, proinflammatory cytokine colon levels). The presence of an Nrf2 inhibitor (ML385) blocked the therapeutic effects of BMSCs. Treatment with BM‐MSCs also exhibited antioxidant and antiapoptosis activities in colon tissues. ML385 reversed all benefits produced by MSCs treatment, suggesting the involvement of Nrf2/Keap1/ARE signaling pathway in MSC actions.	[301]
TNBS‐induced acute colitis	Sprague–Dawley rats	No presensitization, TNBS enema (Day 0)	EV from rat BM‐MSCs	IV	One treatment 3 days after enema	Day 7 after enema	50 µg or 100 µg or 200 µg EV proteins/rat	Modulation of inflammatory, suppression of oxidative stress and alleviation of apoptosis	BMSC‐EVs delivered at higher doses (100 and 200 µg) alleviated DAI and histological score. Furthermore, they reduced mRNA and protein expressions of TNF‐α, iNOS, and COX‐2 in colon tissues. In addition, the higher dose (200 µg) showed antioxidant effects in terms of reduced expression of pro‐oxidant agents (MPO, MDA) and increased expression of antioxidant defense (GSH and SOD). In association, BM‐MSC EV reduced cell apoptosis in colon tissues, indeed lower expression of Caspase‐3, Caspase‐8, and Caspase‐9 (apoptotic markers) were found in treated animals.	[302]
DSS‐induced acute colitis and DSS‐induced chronic and recurrent colitis	C57BL/6 mice	Acute model = DSS for 7 days chronic model = 2 cycles (7 days DSS + 7 days water)	Exosomes from human BM‐MSCs	IV	Acute model = one treatment (Day 2) chronic model = one treatment (Day 7) or twice (Day 7 and Day 16)	Acute model = Day 7 chronic model = Day 28	200 µg Exo proteins/mouse	Macrophage polarization by suppressing NF‐κB activation via metallothionein‐2	MSC‐Exos ameliorated disease related parameters in both acute and chronic colitis models. Exos indeed reduced DAI, histopathological score, and neutrophil infiltration in colon tissues either at Day 7 (acute model) and at Day 28 (chronic model, either after a single treatment and after double treatments). MSC‐Exos administration decreased expression of proinflammatory cytokines (IFN‐γ, IL‐1β, IL‐6, and TNF‐α), whereas increased the expression of the anti‐inflammatory cytokine IL‐10 in colon tissue. Furthermore, MSC‐Exos contribute to the maintenance of intestinal barrier integrity (reduced intestinal permeability and reduced microbial invasion of colonic tissues). MSC‐Exos lost the beneficial effects in DSS‐mice depleted of colonic macrophages (by IP injection of clodronate liposomes). The administration of an antibody anti‐IL‐10 abolished the benefits of MSC‐Exos in colitic mice. Similarly, macrophage depletion impaired the anticolitic effect of MSC‐Exos. Together, these data suggest that the elevated IL‐10 in colons upon MSC‐Exos treatment is potentially derived from colonic macrophages. The knockdown of metallothionein‐2 expression in MSC‐Exos reduced anti‐inflammatory activity of MSC‐Exos by blocking their ability to suppress NF‐κB activation.	[303]
DSS‐induced acute colitis	BALB/C mice	DSS for 7 days	EV from murine BM‐MSCs	IP	Daily treatment for 7 days (during DSS induction)	Day 14	50 µg of EVs/mouse/day	Macrophage polarization via the JAK1/STAT1/STAT6 signaling pathway	EVs attenuated the DSS‐induced colitis in mice in terms of reduced body weight loss, reduced DAI, reduced colon damage score, and MPO activity (neutrophil infiltration) in colon tissue's exerted an anti‐inflammatory effect on DSS‐induced mice, the treatment indeed increased colon levels of M2 macrophages (CD 163+), reduced serum levels of proinflammatory cytokines (IFN‐γ, IL‐12, and TNF‐α) and increased IL‐10 and TGF‐β levels. Moreover, EV treatment attenuated the expression of phospho‐JAK1 and phospho‐STAT1, which is indicative of anti‐inflammatory response while upregulated the expression of phospho‐STAT6 that can be related to IL‐4, a cytokine that triggers the alternative activation of macrophages to the M2 state.	[304]
DSS‐induced acute colitis	C57BL/6 mice	DSS for 8 days	Human adipose‐derived MSCs or MSC‐derived extracellular vesicles (EVs)	IP	One treatment (Day 4)	Day 8	10 × 10^6^ MSCs/mouse EV from supernatants of 10 × 10^6^ MSCs/500 µL PBS	Anti‐inflammatory action via JAK signaling modulation	MSCs and MSC‐derived EVs showed equivalent effects in ameliorating colitis in a murine DSS‐induced model. Both treatments reduced BW loss, prevented colon shortening, and reduced histological damage score. In addition, they reduced proinflammatory cytokine (IL‐6, TNF‐α, IFN‐γ, IL‐17, IL‐12) levels either in serum and in colon tissues of treated mice. IL‐10 levels were instead increased. Moreover, MSC‐ and EV‐treated mice showed reduced colonic levels of phospho‐JAK1 and phospho‐JAK2.	[305]
DSS‐induced acute colitis	C57BL/6 mice	4 days DSS + 3 days water	Exosomes derived from murine olfactory ecto‐MSCs	IV	Twice (on Days 2 and 4)	Day 8	60 µg (total proteins) exosomes /mouse	Modulation of Th1/Th17 and Treg	Exos‐treated mice showed higher colon length and lower disease activity and histological score with respect to control mice. The relative amount (%) of Th1 and Th17 cells were reduced and frequency of Treg cells was increased in MLNs of treated mice.	[306]
DSS‐induced acute colitis	C57BL/6 mice	DSS for 5 days + water for 3 days	Syngeneic BM‐MSCs	IP, IV, or AI (anal injection)	One treatment (Day 5)	Day 8	1 × 10^6^ MSCs/mouse 1 × 10^6^ MSCs in 200 µL diluted Matrigel for AI	Anti‐inflammatory effects possibly via TSG‐6	IP injection showed the highest anticolitic effects in terms of colon length, histologic score and neutrophilic infiltration (MPO activity in colon tissues). MSC treatment reduced mRNA and protein levels of TNF‐α in colon tissues, while increased those of IL‐10. In association MSC administration, and mainly by IP injection, increased serum levels of TSG‐6. Moreover, MSC administration promoted cell proliferation (Ki67‐positive cells) in intestinal crypts and enhanced colonic levels of FoxP3‐positive cells.	[308]
DSS‐induced acute colitis	BALB/c mice	DSS for 7 days + water for 8 days	Syngeneic BM‐MSCs	IV	Three treatments (Days 2, 5, 8)	Day 15	1 × 10^6^ MSCs/dose	Anti‐inflammatory effects	MSC administration improved clinical and histological scores in colitic mice. MSCs reduced serum levels of TNF‐α and reduced mRNA expression of TNF‐α and IL‐1β in colon tissues.	[309]
DSS‐induced acute colitis	C57BL/6 mice	DSS for 7 days + water for 1 day	Syngeneic adipose‐MSCs	IV or IP	Two treatments (Days 2, 5)	Day 8	1 × 10^6^ MSCs/dose	Anti‐inflammatory effects	IV infusion of MSCs was more effective than IP infusion in reducing the clinical and histopathologic severity of colitis. Moreover, IV infusion of MSCs showed a trend in downregulating proinflammatory cytokines (IL‐6, TNF, IL17) and upregulating the anti‐inflammatory ones (IL‐10, IL‐4). However, after IV injection of MSCs increased levels of IFN.	[310]
DNBS‐induced acute colitis	Sprague–Dawley rats	DNBS intrarectal infusion (Day 0)	Syngeneic BM‐MSC sheets or syngeneic adipose‐MSC‐sheets	Endoscopic transplantation	One treatment (4 days after DNBS)	Days 5 and 7	Adipose MSCs: 8.0 × 10^5^ cells/dish BM‐MSCs: 1.1 × 10^6^ cells/dish	Not investigated	MSC sheets application did not ameliorate body weight and DAI in colitic rats. However, treatments reduced ulcer size and endoscopic score (MEICS of lesions).	[311]
TNBS‐induced acute colitis	BALB/c mice	TNBS intrarectal infusion (Day 0)	Murine adipose MSCs or murine BM‐MSCs	IP	One treatment (12 h after TNBS enema)	Day 8	1 × 10^5^ MSCs/mouse	Anti‐inflammatory effects	BM‐MSCs and AD‐MSCs exerted similar therapeutic effects in colitic mice. Both treatments increased survival of colitic mice, reduced disease and histological scores, decreased mRNA and protein levels of proinflammatory cytokines (TNF‐α, IL‐12, and VEGF), while increased the expression of IL‐10 in colonic tissues.	[312]
DSS‐induced acute colitis	C57BL/6 mice	DSS for 7 days	Human adipose MSCs	IV	One treatment: Early treatment (Day 3) or late treatment (Day 11)	Day 11 and Day 21	1 × 10^6^ MSCs/mouse	Promotion of polarization toward regulatory immune cells (Treg and M2 macrophages)	On Day 11, mice early (Day 3) injected with MSCs, showed improved DAI and histological score. Benefits derived from early treatment were maintained also up to Day 21. Early treatment maintained the ability on Day 21, to reduce mRNAs for proinflammatory factors Il‐6, Il‐17a, and TNFα and to increase the mRNAs for M2 macrophage‐related markers CD206 and PGE2 and growth factors VEGF and HGF. In contrast, late treatment (Day 11) did not improved colitis parameters, nor changed mRNA colon levels of proinflammatory cytokines. When MSCs were administered on Day 3 and mice were sacrificed on Day 11, a reduced infiltration of T cells, an increased % of Treg and increased % of CD206+ macrophages were observed in colon tissues.	[314]
DSS‐induced acute colitis	C57BL/6 mice	DSS for 7 days	Human adipose MSCs or CM or umbilical cord‐MSCs or CM	IV	One treatment MSCs (early treatment: Day 3; late treatment: Day 7) Two treatments CMs (Days 3, 4)	Day 11 and Day 21	1 × 10^6^ MSCs/mouse 250 µL CM/dose	Anti‐inflammatory effects and maintenance of a normal microbiota	Early injection (Day 3) of both, AD‐MSCs and UC‐MSCs, improved DAI, colon length, histological score and reduced colonic mRNA levels of TNF‐α at Day 11. The benefits were also observed at Day 21, when early treatments were able to reduce mRNA levels of IL‐6 and IL‐17a and also of IL‐10. When MSCs were late administered, they did not ameliorate colitis and inflammatory indexes. CMs from both MSCs had some therapeutic effects, but reduced compared with injection of MSCs directly. MSC treatment also tended to maintain normal intestinal microbiota.	[315]
DSS‐induced acute colitis	C57Bl/6 mice	5 days	Murine BM‐MSCs primed with IFN‐γ and poly(I:C)	IP	Twice (on Days 1 and 3)	Day 9	3 × 10^6^ MSCs/each dose	MSCs increased Treg cells in inflamed colon tissues in an IDO1‐dependent manner.	Primed MSCs were more effective than unstimulated MSCs to ameliorate the DSS‐induced colitis in mice in terms of body weight, disease activity (DAI), colon length, and histological score. Mice treated with primed MSCs showed higher levels of proliferating (Ki67+) intestinal epithelial cells; increased mRNA expression of stem cell markers (Lgr5 and OLFM4), and of occludin. Moreover, a reduced infiltration of inflammatory cells (monocytes, CD4 T cells, dendritic cells) was observed in spleen and MLN of treated mice. Reduced levels of inflammatory cytokines (IL‐1β, TNF‐α, and IL‐6) and increased levels of IL‐10 were found in colon tissue of treated mice. Primed MSCs MSC expanded Treg cells in the spleen of colitic mice. Interestingly, primed MSCs delivered to mice receiving a competitive IDO inhibitor (L‐1MT) lost their beneficial effects.	[316]
DSS and TNBS‐induced acute colitis	C57BL/6 and BALB/c mice	DSS for 7 days + water for 3 days TNBS presensitization for 7 days + intrarectal TNBS (Day 0)	Exosomes derived from human umbilical cord‐MSCs	IP	DSS model = Three treatments (Days 4, 6, 8) TNBS model = Two treatments (Days 1 and 3)	DSS = Day 10 TNBS = Day 5	300 µg Exo proteins	Promotion of mucosal healing via Wnt signaling pathway	In both models of induced colitis, administration of hUC‐MSC‐exo improved survival, decreased the DAI scores and histopathological score and neutrophil infiltration (MPO activity) in colon tissues. In addition, Exos treatment reduced levels of mRNA expression of IL‐17, IFNg, and TNFa and enhanced those of IL‐10 in colon tissues. With regard to intestinal mucosa integrity, the authors observed reduced intestinal permeability and increased expression of Lgr5 at both mRNA and protein level, suggesting the presence of higher levels of intestinal stem cells in mucosa of mice treated with Exos. By RNA‐seq analysis performed on mRNA extracted from colon tissues of treated and untreated colitic mice, the authors found that Exos treatment promote the activation of Wnt/β‐catenin pathway in colon tissues. The involvement of Wnt/β‐catenin pathway in Exo benefits was supported by the finding that Exo‐treated mice coreceiving a Wnt inhibitor (Wnt‐C59) lost their efficacy in colitis. In particular, Exos lost their ability in regenerating intestinal epithelium.	[317]
TNBS‐induced acute colitis	BALB/c mice	TNBS intrarectal infusion (Day 0)	Human placenta‐derived MSCs embedded or not in chitosan‐based hydrogel with immobilized IGF‐1 C domain peptide	Intracolon injection	One treatment (24 h after TNBS enema)	Day 7	1 × 10^6^ MSCs/mouse	Promotion of macrophage polarization toward an anti‐inflammatory phenotype (M2) possibly via PGE2	Gel‐embedded MSCs were more efficient than MSCs to improve colitis parameters. Treatment reduced BW loss, DAI, and histological score. In addition, gel‐embedded MSCs reduced neutrophil infiltration and activity in colon tissues, reduced ROS activities, and decreased colonic mRNA levels of proinflammatory factors (IL‐1β, IFN‐γ, IL‐6, TNF‐α). Moreover, treatment better maintained epithelial integrity (higher levels of claudin, occludin, and ZO‐1 mRNA). In treated mice, lower levels of M1 macrophages (iNOS+) and higher levels of M2 (CD206+) macrophages were found. The promotion toward M2 polarization was reverted by cotreatment with an inhibitor of EP2 receptors, suggesting a role of PGE2 in promoting macrophage polarization.	[319]
DSS‐induced chronic colitis	C57BL/6J mice	2 cycles (5 days DSS + 5 days water)	Human adipose MSCs and celecoxib (3 mg/kg)	IP	MSCs: twice on Days 6 and 16 celecoxib a COX‐2 inhibitor): for 3 days after MSCs infusions	Day 20	1 × 10^6^ MSCs	Macrophage recruitment	Treatment with adipose MSCs preserved colon lengths and improved the gain in body weight in DSS challenged mice. It also decreased macrophage levels in colon of DSS‐animals, and specifically reduced the M1 (proinflammatory) macrophages. The coadministration of a COX‐2 inhibitor, partially reverted the effects on colonic macrophages, suggesting the involvement of PGE2 in the anti‐inflammatory activity of adipose MSCs. The authors, in in vitro experiments using THP‐1 cells, showed that the anti‐inflammatory activity of PGE2 derived from adipose MSCs is related to PGE2 ability in inhibiting NLRP3 inflammasome formation in THP‐1 cells.	[320]
DSS‐induced acute colitis	C57BL/6J mice	10 days	Exosomes from murine BM‐MSCs primed or not with IFN‐γ	IV	One treatment (on Day 3)	Day 10	200 µg (total proteins) exosomes	Modulation of Th17 and Treg T cells	Treatment with Exos attenuated acute colitis in mice in terms of reduced disease activity index (DAI), histological score. Furthermore, MSC‐Exos Infusion of exosomes reduced Th17 while increased Treg levels (it is not reported where this effect was observed, spleen? MLN? peripheral blood?). Exos derived from primed MSCs were more effective with respect to not Exos from not primed MSCs. In colitic mice, the infusion of miR‐125a and miR‐125b agomir displayed effects similar to those observed after Exos injection. In parallel, miR‐125b agomir injection decreased Th17 cell ratio in colitis mice and slightly increased Treg cell ratio. Since priming with IFN‐γ increased exosome levels of miR‐125a and miR‐125b, the authors suggest that the anti‐inflammatory effects (on Th17 T cells) are mediated by these miRNAs.	[321]
DSS‐induced acute colitis	Balb/c mice	DSS for 10 days	Human umbilical cord‐MSCs	IP	Three doses (Days 3, 6, 9)	Day 9, 10, or 11	3 × 10^6^ MSCs	Regulation of 15‐lox‐1 expression in macrophages through exosomal miR148b‐5p	MSC treatment attenuated DSS‐induced colitis. MSCs reduced body weight loss, reduced DAI and colon lesions. In addition, reduced levels of inflammatory cytokines (IL‐1β, IL‐6, and TNF‐α) were observed in colon tissues of treated animals. When macrophages were removed (by using CD11b‐DTR mice) before or on the day of treatment, the effect of MSCs on colitic mice were impaired. In this study, the authors found that exosomes generated from hUC‐MSCs contained miR148b‐5p and that miR148b‐5p derived from hUCMSCs can downregulate the 15‐lox‐1 expression in macrophages in vitro. The treatment of colitic mice with hUC‐MSCs transfected with a synthetic oligonucleotide complementary to miR148b‐5p (inhibitor) was unable to improve disease parameters. These results indicate that miR148b‐5p from hUC‐MSCs attenuated colitis through downregulating 15‐lox‐1 expression.	[322]
DSS‐induced acute colitis	BALB/c mice	DSS for 11 days	Exosomes from hUC‐MSCs	IV	Three doses (Days 3, 6, 9)	Day 11	1 mg Exosomes (Exo) proteins/dose	Regulation of inflammasome activation in macrophages	The administration of exosomes relieved mouse weight loss and reduced DAI and reduced the expression of inflammatory cytokines (IL‐6, TNF‐α, IL‐10) in mouse colon tissues. Protein and gene expressions of NLRP3 inflammasome‐related molecules (NLRP3, ASC, Caspase‐1, IL‐18, IL‐1β) were decreased in mice treated with Exos. Immunohistochemistry of colon tissue revealed lower NLRP3 synthesis and activation in Exo‐treated mice, with a consequent reduced expression of colonic IL‐1β. In vitro studies showed that exosomes decreased the activation of NLRP3 inflammasomes in peritoneal macrophages and delay the progress of cell pyroptosis. In addition, still in vitro, the authors observed that MSC‐derived exosomal miR‐378a‐5p block NLRP3 inflammasome assembly and the consequent cleavage of Caspase‐1.	[323]
TNBS‐induced acute colitis	Wistar rats	TNBS enema (Day 0)	Rat adipose‐derived MSCs	IV	One treatment (Day 8)	Day 28	1 × 10^7^ MSCs	Preservation of colon tissue cell integrity possibly via activation of noncanonical Wnt signaling pathway	MSCs treatment ameliorated DAI, reduced apoptosis (TUNEL+ cells and levels of cleaved Caspase‐3) in colon tissues, reduced the expression of proinflammatory cytokine (IFN‐γ, IL‐2, IL‐17, and IL‐23), and increased the expression of anti‐inflammatory cytokine (IL‐4, IL‐13, IL‐10, and TGF‐β). Moreover, MSC treatment reduced Th1 and Th17 cells and upregulated Th2 and Treg cells.	[324]
DSS‐induced acute colitis	C57BL/6 mice	DSS from Day 3 to Day 9	Exosomes from human BM‐MSCs and Exos from LPS‐primed hBM‐MSCs and Exos from MSCs transfected with miR‐181‐inhibitor	IV	Four treatments (Days 1, 3, 5, and 7)	Day 9	32 mg Exo proteins/kg mouse	Improvement of gut microbiota composition	Exos from LPS‐primed MSCs were more effective in ameliorating mice colitis with respect to Exos from not primed MSCs. Treatments with Exos more preserved colon length, reduced TUNEL+ cells in colon tissues, reduced serum levels of TNF‐α, IL‐6, and IL‐1β. The injection of Exos from MSCs transfected with miR‐181‐inhibitor, did not ameliorate mice colitis and reverted the improvement in mucosal barrier function (increased expression of Claudin‐1 and ZO‐1) observed with treatment with not‐transfected Exos. These data suggest exosomal miR‐181 involvement in Exos benefits. Exos treatment alleviated the microbiota alterations induced by DSS. The expression abundance of Lactobacillus showed an upward trend, while that of Bacteroides showed a downward trend after treatment with MSC‐Exos.	[325]
DSS‐induced chronic colitis	C57BL/6 mice	Three DSS cycles:3x (DSS 1 week + water 2 weeks)	Exos from human umbilical cord MSCs	IV	Nine treatments (Days 3, 10, 17, 24, 31, 38, 45, and 59)	Day 63	1 × 10^9^ particles	Antifibrogenic effect, possibly by inhibiting ERK phosphorylation	Exos administration reduced body weight loss and reduced DAI. Moreover, Exos reduced mRNA expression of fibrosis‐related markers such as TGF‐β, α‐SMA, COL1A1, COL3A1, and FN1. In colon tissues of mice treated with Exos the ratio phospho‐ERK/total‐ERK was decreased. Similarly, in in vitro experiments, the authors found that intestinal fibroblasts treated for 48 h with TGF‐β showed increased p‐ERK/t‐ERK ratio, which was decreased by Exos treatment. The expressions of α‐SMA, fibronectin, and collagen were reduced in fibroblasts treated with a ERK inhibitor (PD98059). These data suggested a possible involvement of this pathway in the fibrogenic process.	[327]
DSS‐induced acute colitis	C57BL/6 mice	DSS for 7 days + water for 2 days	Human umbilical cord MSCs	IV	Two treatments (Days 3 and 5)	Day 9	1 × 10^6^ MSCs/dose	Improvement of gut microbiota composition	MSC administration significantly ameliorated DSS‐induced colitis in terms of reduced weight loss, decreased mortality, lower DAI and histopathological score. MSC treatment reduced serum levels of IL‐1β, TNF‐α, and IL‐6, reduced relative levels of CD3+ cells in peripheral blood and reduced CD3+ cells, and Ly6G cells in spleens. The relative abundances of probiotics (*Firmicutes*, *Blautia*, *Roseburia*, *Helicobacter*, and *Lactobacillus*) were higher in the microbiota of treated mice. MUC‐1 mRNA was found upregulated in colonic tissues of treated mice.	[328]
Radiation‐induced colorectal fibrosis	eGFP transgenic Sprague–Dawley rats	A single dose of 29 Gy from X‐ray source (2.43 Gy/min), delivered to a 2 × 3 cm colorectal area	Rat adipose MSCs or MSCs silenced for HGF (siHGF‐MSCs) or MSCs silenced for TSG6 (siTSG6‐MSCs)	IV	Four doses (twice weekly at 2 and 3 weeks after irradiation during initiation of fibrosis)	During the first phase of fibrosis (4 weeks), intermediate phase (5 weeks) and the phase of established fibrosis (7 weeks) after the irradiation	5 × 10^6^ MSCs/dose	Antifibrogenic effect, possibly via HGF and TSG6	Seven weeks postirradiation, mucosal ulceration, edema in submucosa stimulated by ECM accumulation, and overall thickening were observed, in association with upregulation of the expression of extra cellular matrix components, matrix metalloproteinases (MMPs), serpine‐1 (PAI‐1), and tissue inhibitors of metalloproteinases (TIMPs). MSC treatment reduced mRNA expression of ECM molecules (collagen, SPARC, CTGF) and increased colon levels of MMP2. HGF and TSG‐6 silencing in transplanted MSCs resulted in a significant increase in ECM deposition in colon suggesting the involvement of these factors the pathophysiology of intestinal fibrosis.	[329]
DSS‐induced acute colitis	C57BL/6 mice	DSS for 7 days + water for 3 days	Human umbilical cord MSCs	IP	One treatment (Day 5)	Day 10	1 × 10^6^ MSCs/mouse	Amelioration of DSS‐induced dysbiosis, possibly by modulating the Tregs–IgA axis	MSC administration significantly ameliorated DSS‐induced colitis in terms of reduced weight loss, decreased mortality, lower DAI and histopathological score. In addition, MSC treatment reversed microbiota alterations induced by DSS, and specifically decreased Firmicutes and increased *Bacteroidota*. The authors also established, by LEfSe analysis, that MSC treatment downregulated the type of taxa that are dominant in gut microbiota dysbiosis induced by DSS. MSC treatment increased levels of Treg in Peyer's patches, small intestine lamina propria, colonic lamina propria and mesenteric lymph nodes and increased IgA+ B cells in the two last districts. IgA expression in small intestine tissue was higher in MSC‐treated mice as well as the proportion of IgA‐coated bacteria in stool pellets.	[330]
DSS‐induced acute colitis	C57BL/6 mice	DSS for 7 days (from Day 0 to Day 7) + water for 3 days	Human umbilical cord MSCs ± broad‐spectrum cocktail of antibiotics = ABX (for gut microbiota depletion)	IP	MSCs = one treatment (Day 5) ABX = daily for 5 days (Days −5 to Day 0)	Day 10	1 × 10^6^ MSCs/mouse	MSCs act on colitis through a microbiota‐dependent mechanism and possible link between MSC anti‐inflammatory action and microbiota‐derived short‐chain fatty acids production.	MSC administration significantly ameliorated DSS‐induced colitis in terms of reduced weight loss, decreased mortality, lower DAI and histopathological score. Increased % of Treg and Th2 cells and reduced % of Th17 T cells were observed in colonic lamina propria and mesenteric lymph nodes of treated mice. MSC treatment reversed intestinal dysbiosis induced by DSS, decreasing *Bacteroidota, Proteobacteria*, and increasing *Firmicutes*. The authors observed that the benefits of MSCs on clinical and colitis inflammation were abrogated by antibiotic treatment while, instead the transplantation of feces from MSC‐treated mice into ABX‐mice re‐established disease ameliorations.	[331]

For each study, the table reports the inflammation model and inducing agent, animal strain, therapeutic intervention with cell source, dose and administration schedule, and the principal disease‐activity, histological, immunological, and (where assessed) fibrosis outcomes.

Abbreviations: FMT, fecal microbiota transplantation; MSCs, mesenchymal stromal cells; DSS, dextran sulfate sodium; DNBS, 2,4‐dinitrobenzenesulfonic acid; TNBS, 2,4,6‐trinitrobenzene sulfonic acid; CM, conditioned medium; EVs, extracellular vesicles; Exos, exosomes; IV, intravenous; IP, intraperitoneal; OS, oral; SC, subcutaneous; MCP1, monocyte chemoattractant protein‐1; IFN, interferon; AhR, aryl hydrocarbon receptor; SPF, specific pathogen‐free; TGF, transforming growth factor; RAG, recombination activating 1 gene; APC, antigen presenting cells; EGFP, enhanced green fluorescent protein; CCL, C–C motif chemokine ligand; CXCR, C–X–C motif chemokine receptor; DAI, disease activity index; mRNA, messenger ribonucleic acid; BW, body weight; MUC, mucin; THBS1, thrombospondin‐1; MPO, myeloperoxidase; GAS, growth arrest‐specific protein; IDO, indoleamine 2,3‐dioxygenase; MLN, mesenteric lymph node; FP, fetal placenta; PHA, phytohemagglutinin; IL1‐Ra, IL‐1 receptor antagonist; NK, natural killer; CXCL, C–X–C motif chemokine ligand; iPSC, induced pluripotent stem cell; TSG‐6, tumor necrosis factor‐stimulated gene‐6; Smoc2, SPARC‐related modular calcium‐binding protein 2; Lgr5, leucine‐rich repeat‐containing G‐protein coupled receptor 5; CBC, crypt base columnar; HA, hyaluronic acid; Fizz1, resistin‐like beta 1; Ym1, chitinase 3‐like 3; Nrf2, nuclear factor erythroid 2‐related factor 2; KEAP1, Kelch‐like ECH‐associated protein 1; ARE, antioxidant response element; ZO‐1, zonula‐occludens 1; ROS, reactive oxygen species; MDA, malondialdehyde; GSH, reduced glutathione; SOD, superoxide dismutase; COX2, cyclooxygenase‐2; NF‐kB, nuclear factor kappa‐light‐chain‐enhancer of activated B cells; JAK, Janus kinase; STAT, signal transducer and activator of transcription; PBS, phosphate buffered saline; MEICS, murine endoscopic index of colitis severity; iNOS, inducible nitric oxide synthase; EP2, prostaglandin E2 receptor 2; PGE2, prostaglandin E2; VEGF, vascular endothelial growth factor; HGF, hepatocyte growth factor; OLFM4, olfactomedin4; NLRP3, NLR family pyrin domain containing 3; THP1, Tohoku Hospital Pediatrics‐1; 15‐LOX1, 15‐lipoxygenase‐1; Wnt, wingless‐type MMTV integration site family; LEfSe, linear discriminant analysis effect size; TUNEL, terminal deoxynucleotidyl transferase dUTP Nick‐end labeling; ERK, extracellular signal‐regulated kinase; COL, collagen; FN, fibronectin; SMA, smooth muscle actin; SPARC, secreted protein, acidic and rich in cysteine; CTGF, connective tissue growth factor.

**TABLE 4:** Clinical studies of emerging nonimmune‐targeted therapies for inflammatory bowel disease.

Pathology indication	Treatment	Delivery route	Pivotal trials (registration no. at www.clinicaltrials.gov)	Trial phase	Study duration	Authors conclusions	References
Moderate‐to‐severely active UC	ABX464 (obefazimod)	OS	NCT03093259 and NCT03368118	Phase IIa	2017–2020	Induction study (8 weeks of treatment): obefazimod more effective than placebo in achieving endoscopic improvement and reduction of the MCS and placental MSC‐(pMCS). Maintenance study: sustained remission and additional patients into remission	[244]
Moderate‐to‐severely active UC	ABX464 (obefazimod)	OS	NCT04023396 (long‐term study)	Phase IIb	2017–2024	Sustained efficacy and safety of obefazimod 50 mg once daily, with a significant number of patients reaching clinical remission at Weeks 48 and 96. Improvements in endoscopy scores and fecal calprotectin (FCP) levels. Limitation: absence of a control group	[245]
Moderate‐to‐severely active UC	ABX464 (obefazimod)	OS	NCT05507203, NCT05507216, NCT05535946	Phase III (double‐blind, randomized, placebo‐controlled Phase 3)	Ongoing	The results have not yet been published.	No published results

Mild to moderate active UC	Fecal microbiota transplantation: pooled donor stool FMT (dFMT) or autologous FMT (aFMT)	Enema	ACTRN12613000236796	Not indicated	2013–2017	1‐week treatment with anaerobically prepared donor FMT compared with autologous FMT resulted in a higher likelihood of remission at 8 weeks.	[246]
Active UC without infectious diarrhea	FMT (50 mL, from healthy anonymous donors)	Enema	NCT01545908	Phase II	2012–2014	FMT induces remission in a significantly greater percentage of patients with active UC than placebo (24% with FMT versus 5% with placebo).	[247]
Active ulcerative colitis (Mayo score 4–10)	Multidonor FMT (150 mL)	Enema	NCT01896635	Phase II	2013–2015	Intensive‐dosing (40 infusions over 8 weeks), multidonor, fecal microbiota transplantation induces clinical remission (27% of FMT‐treated patients compared with 8% in controls) and endoscopic improvement in active ulcerative colitis and is associated with distinct microbial changes that relate to outcome.	[248]
Mild to moderately active UC	FMT (500 mL) from healthy donors or autologous fecal microbiota (control)	Nasoduodenal infusion	NCT01650038	Phase II	2012–2014	No statistically significant difference in clinical and endoscopic remission between patients with UC who received fecal transplants from healthy donors and those who received their own fecal microbiota	[249]
Moderate to severe UC (Mayo 4–10)	FMT (allogenic or autologous) 500 mL FMT preparations with standardized cell density of 10 [10] cells/mL	Enema	NCT03110289 (RESTORE‐UC trial)	Not reported	2017–2021	The study did not meet its primary endpoint of increased steroid‐free clinical remission at Week 8.	[262]

Refractory Crohn's disease	Autologous bone marrow MSCs (1–2 × 10^6^ cells/kg body weight)	IV	NTR1360	Phase I	Not reported	Administration of autologous bone marrow‐derived MSCs appears safe and feasible in the treatment of refractory Crohn's disease.	[332]
Crohn's disease	Autologous Bone marrow MSCs (2, 5, or 10 million cells/kg BW)	IV	NCT01659762	Phase I	2012–2015	All patients tolerated MSC infusion without dose‐limiting toxicity. Seven had serious adverse events, including five hospitalizations for Crohn's disease flare, with two possibly related to the infusion.	[333]
Crohn's fistula	Autologous adipose tissue MSCs (1–4 × 10^7^ cells)	Intrafistula	not reported	Phase I	2008–2009	Adipose stromal cells (ASCs) were well tolerated and demonstrated a potential for the treatment of Crohn's fistula.	[334]
Refractory rectovaginal Crohn's fistulas	Autologous adipose‐derived MSCs on a bioabsorbable scaffold (MSC‐MATRIX)	Intrafistula	not reported	Phase I	Not reported	Surgical placement of an autologous adipose‐derived MSC‐coated fistula plug in diverted patients with Crohn's rectovaginal fistulas was safe and feasible. All patients had a reduction in the size of their fistula tract, and 3 of 5 had cessation of drainage, but none achieved complete healing.	[335]
Perianal fistulas in patients with CD	Autologous adipose tissue derived MSCs	Intrafistula	NCT03803917	Not applicable	2015–2018	Adipose‐MSC injections found to be safe and to result in complete fistula healing in 57% of patients. Two patients developed small abscesses, 1 had urinary retention, and 1 had minor bleeding during liposuction. (Study performed on 21 total patients).	[336]
Perianal fistulas in patients with Crohn's disease	Matrix‐loaded adipose autologous MSCs (20 × 10^6^ cells per plug)	Intrafistula placement of MSC‐MATRIX	NCT01915927	Phase I	2013–2019	At 6 months, 10 of 12 patients (83%) had complete clinical healing and radiographic markers of response.	[337]
Moderate to severe CD (CDAI] between 220 and 450)	Allogeneic umbilical cord‐MSCs (1 × 10^6^ UC‐MSCs/kg)	IV	NCT02445547	Phase I–II	2012–2015	Twelve months after treatment, patients in umbilical cord‐MSC (UC‐MSC) group showed higher CDAI and HBI, and lower corticosteroid dosage with respect to control patients.	[338]
Active luminal CD refractory to biologic therapy (CD activity index [CDAI], >250)	Allogeneic bone marrow‐derived MSCs (2–2.7 × 10^6^ cell/kg)	IV	NCT01090817	Phase II	2010–2015	4 infusions of 2 × 10 [6] cell/kg at weekly intervals led to clinical response in 12 patients (80%), clinical remission in 8 patients (53%), and endoscopic improvement in 7 patients (47%).	[339]
Refractory perianal fistulising Crohn's disease	Allogeneic bone marrow‐derived MSCs (1–9 × 10^7^ cells)	Intrafistula	NCT01144962	Phase I–II	2012–2018	Local bone marrow‐MSC (BM‐MSC) therapy for perianal CD fistulas was maintained for up to 4 years after treatment. Specifically, 75% [3/4] and 100% (4/4) of the patients treated with 1 × 10^7^ and with 3 × 10^7^, respectively, had complete clinical fistula closure after 4 years. In the three patients in the placebo group, none of the patients experienced partial or complete fistula closure at 4 years.	[340]
CD patients with symptomatic nonpassable stricture, less than 5 cm in length, refractory to conventional or biologic therapies	Allogeneic bone marrow‐derived MSCs (3 × 10^7^ cells)	Intrafistula		Phase I–II	2018–2020	Local MSC injection in nonpassable CD strictures was well tolerated over the short term, although several occlusions occurred in the follow‐up, indicating insufficient therapeutic effect in those cases. The efficacy of MSC injection in the strictures remains difficult to interpret in this study in absence of a control group.	[341]
Severe refractory luminal CD	Allogeneic bone marrow‐derived MSCs (1.5–2 × 10^6^ cell/Kg BW)	IV	NCT01540292	Phase I–II	2012–2014	Decrease in CDAI at Week 8 was observed in 8 patients, but only 2 reached the primary endpoint of clinical response at Week 8, among whom 1 achieved clinical remission. Two other patients achieved clinical remission at Weeks 4 and 12, respectively. CDAI, CRP, and fecal calprotectin levels were not significantly impacted by the treatment.	[342]
Refractory perianal Crohn's disease fistulas	Exosomes from cultured allogeneic umbilical cord‐MSCs	Intrafistula	NCT05499156	Phase I	2022	None of the 5 patients developed acute or latent complications and reactions. No control group.	[343]

For each trial the table reports the IBD indication (UC, CD, or complex perianal CD), the therapeutic product and cell or molecular source, route and schedule of administration, trial phase and registration identifier, and the principal published clinical, endoscopic, and safety outcomes (or the expected outcomes for trials still in progress).

*Abbreviations*: FMT, fecal microbiota transplantation; MSCs, mesenchymal stromal cells; OS, oral; IV, intravenous; FCP, fecal calprotectin; ASCs, adipose stromal cells; CRP, C reactive protein, CDAI, Crohn's disease activity index; HBI, Harvey–Bradshaw index.

#### 3.2.1 miRNA‐124 Upregulators

MicroRNAs (miRs) are small noncoding RNA oligonucleotides that regulate the expression of a large number of genes and are centrally involved in the pathogenesis of different human inflammatory diseases [239]. Studies have shown that certain miRs are deregulated in IBD, specifically miR‐124, leading to increased levels of STAT3 expression and the transcription activation of its downstream targets [240]. STAT3 is known to be upregulated in UC and has also been implicated in the progression of UC to colon cancer [241].

**FIGURE 3:**
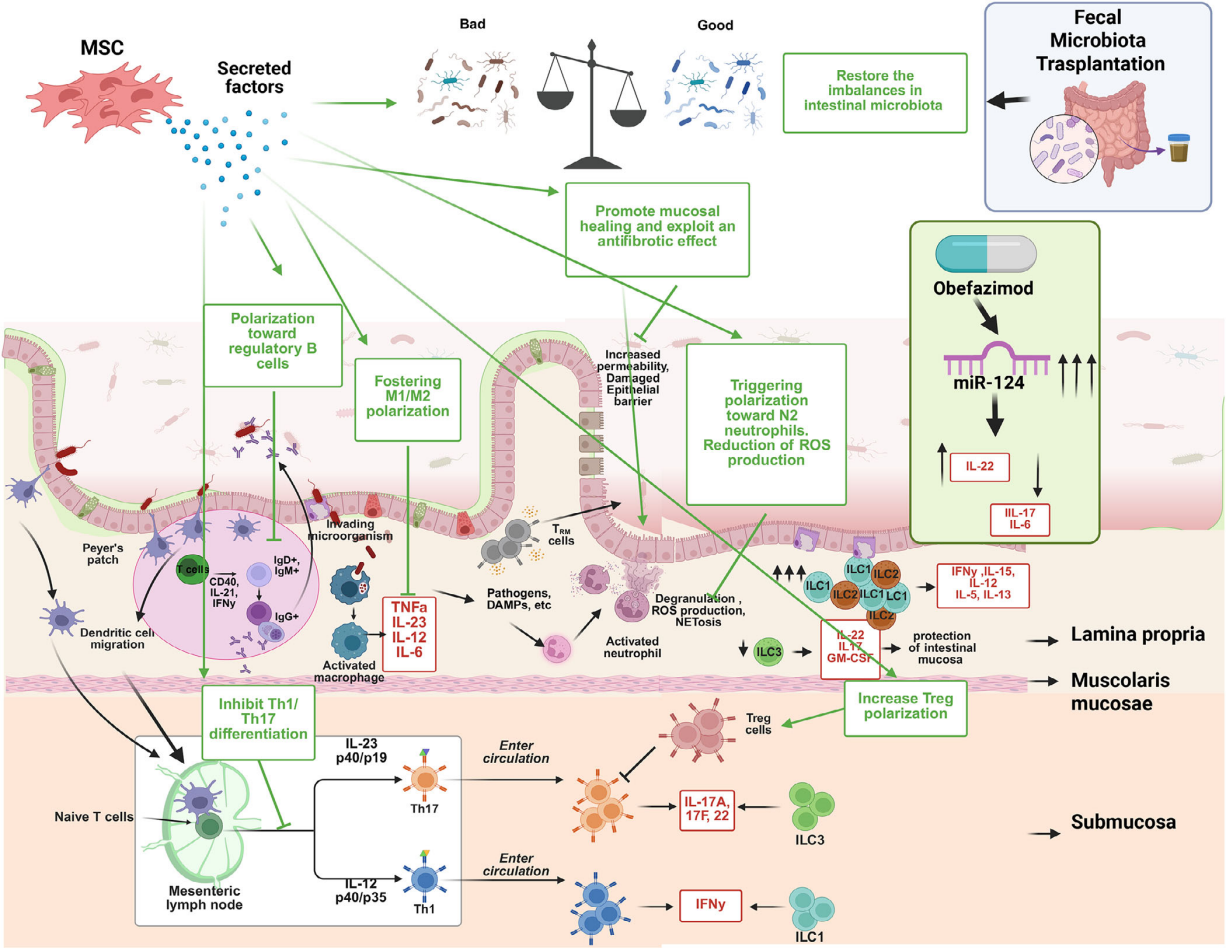
Biologic therapies for inflammatory bowel disease. Biologic agents, particularly those targeting TNF‐α, remain the cornerstone of current therapeutic strategies in inflammatory bowel disease (IBD), leading to significant improvements in clinical outcomes. However, their efficacy is limited by primary nonresponse and secondary loss of response in a substantial proportion of patients. Recently developed biologics targeting interleukins (IL‐12, IL‐23) and lymphocyte trafficking pathways have shown encouraging results in addressing these limitations. Nonetheless, a considerable subset of patients continues to progress to surgery due to refractory disease. In response, emerging therapeutic approaches are being explored to address the complex, multifactorial nature of IBD. These include multitargeted biologics, mesenchymal stromal cell (MSC)‐based therapies, and interventions beyond immune modulation, such as fecal microbiota transplantation (FMT) and novel small molecules like obefazimod, which acts by enhancing the expression of microRNA‐124 (miR‐124), a key regulator of inflammatory pathways. Together, these strategies aim to expand the therapeutic landscape by targeting diverse pathogenic mechanisms underlying IBD.

The therapeutic induction of miRNA‐124 has emerged as a novel approach for treating UC (Figure 3; see Table 3). An initial experimental study demonstrated that treatment with ABX464, later named obefazimod, significantly reduced DSS‐induced colitis in mice by lowering colonic inflammatory cytokines and increasing the anti‐inflammatory IL‐22 expression in macrophages [242]. Later, obefazimod was found to raise miR‐124 levels, which in turn decreased proinflammatory cytokines such as IL‐17 and IL‐6, both in an IBD mouse model and in patients with UC [243]. Furthermore, obefazimod has shown safety and efficacy in patients with moderate‐to‐severely active UC during a Phase IIa induction trial [244] (see Table 4). A subsequent Phase IIb study [245] showed that in patients with moderate‐to‐severely active UC, 50 mg obefazimod was effective in improving patients’ condition at Week 48 and sustained this efficacy through Week 96. At Week 96, 52.5% of patients achieved clinical remission, closely mirroring the 54.8% remission rate observed at Week 48. Endoscopic improvements were similarly sustained, with 59.0% of patients showing improvement at Week 96 versus 61.3% at Week 48. The sustained long‐term efficacy of obefazimod corresponds with a 2‐year increase in miR‐124 expression in both blood and rectal tissue. The ongoing Phase 3 ABTECT program—a double‐blind, randomized, placebo‐controlled trial (NCT05507203, NCT05507216, NCT05535946)—is further evaluating the safety and effectiveness of obefazimod in UC patients.

#### 3.2.2 Fecal Microbiota Transplantation

FMT has demonstrated the capacity to induce remission in three [246, 247, 248] of four randomized controlled trials in UC [249] (Figure 3). However, its therapeutic application remains constrained by the absence of standardized protocols, the modest and often transient nature of clinical benefit, and ongoing safety concerns. In particular, the use of FMT in patients with IBD complicated by *Clostridioides* difficile infection is controversial, and the US FDA has issued safety alerts citing risks of transmission of multidrug‐resistant organisms (https://www.fda.gov/safety/medical‐product‐safety‐information/fecal‐microbiota‐transplantation‐safety‐communication‐risk‐serious‐adverse‐reactions‐due, 2019) and pathogenic microbes (https://www.fda.gov/vaccines‐blood‐biologics/safety‐availability‐biologics/safety‐alert‐regarding‐use‐fecal‐microbiota‐transplantation‐and‐risk‐serious‐adverse‐events‐likely, 2020).

To overcome these limitations, current research is investigating defined microbial consortia, microbial metabolites, and prebiotic interventions, though results to date remain heterogeneous [246, 247, 248, 249]. A key unresolved question is the identity of the active microbial or metabolic components within donor stool that confer therapeutic benefit, which hampers rational therapeutic development.

Numerous FMT studies, where fecal samples from IBD patients are transplanted into germ‐free murine models, provide powerful tools for elucidating microbiota–host immunity interactions [250]. Approaches have ranged from testing single microbial species to transplanting complex microbial communities (see Table 3). For example, fecal microbiota from patients with IBD increases colitis susceptibility in germ‐free mice, associated with enhanced Th17 responses and diminished Treg activity [251]. Another study involving germ‐free mice transplanted with stool from CD mothers or their 3‐month‐old infants demonstrated that the dysbiosis associated with maternal IBD, marked by decreased levels of Treg and IgA^+^ B cell subsets, can be transmitted to the offspring [252]. Progress in this field includes the identification of 17 *Clostridia* strains by Atarashi and colleagues that promote Treg differentiation via TGF‐β‐dependent mechanisms, thereby ameliorating experimental colitis [253].

Other experimental studies evidenced that transplanting fecal microbiota from healthy normobiotic mice ameliorate disease activity index (DAI) and colon histology in mice with DSS‐induced colitis (see Table 3). This disease amelioration is linked to a restoration of a healthy gut microbiome in colitic mice [254, 255, 256], associated with a reduction of the proinflammatory profile of host immunity. In this regard, FMT has been shown to reduce levels of colonic T cells [254], promote the production of tolerogenic, anti‐inflammatory IL10 by mucosal innate and adaptive cell immune populations [255], modulate the IgA/G‐mediated immune response [257], and promote intestinal metabolite production, such as indole‐3‐acetic acid and tryptophan, which acting as ligands for AhR, regulate the AhR signaling pathway to suppress inflammation [258].

Furthermore, transplanting healthy donor‐derived microbiotas into germ‐free mice colonized with microbiotas from human donors with CD, increased the levels of RORγt^+^ Treg cells while suppressed those of gut Th17 cells [259]. Interestingly, the authors identified a specific strain of E. coli that induces Th17‐driven inflammatory responses in mice and showed that it is impaired by FMT [259]. More recently, *B. thetaiotaomicron* and *F. prausnitzii* have been identified as the key bacteria in donor feces able to alleviate colitis in mice via increasing lecithin and regulating IL‐10 production of intestinal Tregs [260].

Clinical trials in UC are now underway (see Table 4), and advances in microbial engineering may allow precise tailoring of strains for therapeutic purposes [261]. Although current evidence suggests FMT can achieve modest benefit in carefully selected UC patients [246, 248], its overall efficacy is limited and does not support adoption as standard care. Patient selection, donor material characterization, and rigorous protocol standardization remain critical unmet needs [262]. Encouragingly, recent developments include standardized frozen FMT preparations and encapsulated formulations [263], which may enhance feasibility and reproducibility. To firmly establish efficacy, durability of response, and long‐term safety, large‐scale, multicenter randomized controlled trials employing standardized methodologies are urgently required.

#### 3.2.3 MSCs and Their Secretome

Despite the expanding therapeutic armamentarium for IBD, an optimal treatment strategy remains elusive. Approximately 11% of patients with UC and up to 70% of patients with CD ultimately require surgery owing to refractory disease or complications [264].

The multitargeted approach based on the combination of multiple biologics with different mechanisms of action represents an emerging strategy. Combination therapy with biologics targeting distinct immune pathways represents an emerging approach, yet the complex and multifactorial nature of IBD pathogenesis underscores the need for strategies that extend beyond immune modulation alone. In this regard, adult stem cell‐based therapies, particularly those utilizing MSCs, have gained interest due to their dual immunomodulatory and regenerative properties, which may both attenuate inflammation and promote mucosal repair [265].

MSCs can be isolated from diverse tissue sources, including bone marrow, adipose tissue, umbilical cord, and other perinatal tissues, and are defined according to criteria established by the *International Society for Cellular Therapy* [266, 267]. Beyond direct cell transplantation, increasing attention has focused on the MSC secretome, that comprises soluble mediators and extracellular vesicles (EVs), such as exosomes, which may mediate many of the therapeutic effects attributed to MSCs and represent a promising avenue for cell‐free therapy in IBD [268]. Studies have demonstrated that MSC‐derived secretome, cultivated from cell cultures, ameliorates pathophysiological parameters in animal models of IBD with comparable efficacy to direct MSC transplantation [269, 270]. The cell‐free nature of the secretome offers notable advantages, including simplified administration, reduced risks associated with cell transplantation, and potentially lower costs. Exploiting these paracrine signaling and immunomodulatory properties, researchers aim to formulate novel cell‐free therapeutic strategies for IBD, harnessing the regenerative potential of these bioactive molecules [271, 272]. Further optimization of secretome production, standardization, and delivery methods, alongside elucidation of precise mechanisms, remains critical for clinical translation [273].

##### 3.2.3.1 Preclinical evidences

Preclinical evidence strongly supports MSCs and their secretome as promising therapeutic tools for IBD, attributed to their multifaceted activities, including immunomodulatory, antioxidant, antiapoptotic, antifibrotic, regenerative, angiogenic, and antimicrobial effects [270, 274] (see Table 3). Various MSC sources, including bone marrow (BM‐MSCs) [275, 276, 277, 278, 279, 280], umbilical cord (UC‐MSCs) [281, 282, 283], adipose tissue (AT‐MSCs) [284, 285, 286], amnion‐derived [287], endometrial‐derived (E‐MSCs) [288], and induced pluripotent stem cell‐derived (iPSC‐MSCs) [289, 290], have been investigated. While local MSC injections have demonstrated compelling evidence for efficacy and safety, systemic infusions for luminal IBD remain an active area of investigation [291].

Preclinical studies exploring the efficacy of MSCs and their secretome have been predominantly performed on mouse models of colonic inflammation induced by chemicals like DSS or 2,4,6‐trinitrobenzene sulfonic acid [292].

For Crohn's‐like ileitis, the SAMP1/YitFc mouse model offers a stronger resemblance to the human condition [293, 294]. Crucially, a majority of SAMP1/YitFc mice over 40 weeks exhibit perianal disease and fibrostenotic/stricturing, which are features of human CD not commonly observed in other IBD animal models. To date, only one study has employed this model to investigate MSC efficacy in IBD [280].

Most preclinical studies consistently report that MSC treatment significantly leads to significant improvements in several IBD parameters, such as increased survival rates, reduced DAI, longer colon length, and better histopathological outcomes [280, 295, 296] (see Table 3). Furthermore, these studies commonly observe decreased intestinal infiltration of immune cells, lowered levels of proinflammatory cytokines (e.g., IL‐6, IL‐17, TNF‐α), and increased levels of the anti‐inflammatory cytokine IL‐10 [280, 295, 296]. These beneficial effects are observed in both acute and chronic disease models.

Intriguingly, therapeutic benefits are not strictly dependent on the physical presence of MSCs in intestinal tissues. Positive outcomes have been achieved using cell‐free strategies, such as conditioned medium (CM)/secretome from MSC cultures (CM‐MSC) [288, 297, 298, 299, 300, 301] and EVs or exosomes extracted from CM‐MSC [284, 302, 303, 304, 305, 306].

While the majority of studies report positive results, a limited number show negative or conflicting outcomes. For instance, some studies found syngeneic BM‐MSCs did not improve survival or reduce clinicopathologic severity [275]. Another study reported opposing effects between cell and EV treatments, with BM‐MSCs worsening disease but EVs from primed BM‐MSCs improving it [278].

These discrepancies highlight critical issues for clinical translation, including optimal administration routes, tissue sources, dosages, and timing of administration.

Below, we synthesize the impact of each of these factors on the effectiveness of MSCs and their secretome in preclinical IBD models. Intraperitoneal [307, 308], intravenous [287, 309, 310], and enema [222, 287] routes all show efficacy in chemically induced colitis; endoscopic submucosal injection and retention strategies including hydrogels [288], nanofiber composites [286], and cell sheets [311] enhance local delivery and lesion‐site exposure.

No clear superiority has been demonstrated based on MSC tissue source, with adipose‐, bone marrow‐, and iPSC‐derived MSCs and EVs yielding comparable effects [231, 290, 305, 312].

An optimal dosage for MSCs, CM, or EVs remains undefined across cell types. Dose‐dependent benefits have been reported [281, 285], with typical single doses ranging from 1 to 10 × 10^6^ MSCs per mouse [313].

Early or prophylactic administration sustains benefits even after disease relapse [279, 280, 285, 313]. Delayed dosing often fails to ameliorate acute models [314, 315] but can still reverse established fibrosis [276], indicating that timing must be tailored to the predominant pathological process.

Preclinical investigations have been pivotal in elucidating the multiple mechanisms through which MSCs and their secretome exert therapeutic effects in IBD (Table 3).

Preclinical data identify immunomodulation of T‐cell and macrophage responses as the therapeutically dominant mechanism by which MSCs ameliorate IBD. MSCs and MSC‐derived EVs suppress proinflammatory Th1 and Th17 cells and expand Tregs in intestinal tissues, spleen, and mesenteric lymph nodes [231, 281, 284, 285, 306, 316], and polarize macrophages toward an anti‐inflammatory M2 phenotype [280, 296, 303, 304, 317, 318]. These effects are largely paracrine, mediated by indoleamine‐2,3‐dioxygenase [281, 316], prostaglandin E_2_ [319, 320], TNF‐stimulated gene‐6 (TSG‐6) [295, 296], and EV‐cargo miRNAs (miR‐125a/b, miR‐148b‐5p, miR‐378a‐5p) [321, 322, 323], which collectively dampen macrophage expression of 15‐lipoxygenase‐1 [322] and NLRP3‐inflammasome activation [323] and reduce proinflammatory cytokine production (IL‐6, IL‐17, TNFα) while increasing IL‐10.

MSC‐derived exosomes and soluble factors also promote intestinal mucosal healing by enhancing survival and proliferation of Lgr5+ intestinal stem cells [290, 317, 324], upregulating tight‐junction proteins (ZO‐1, occludin, claudin‐1) [289, 325, 326], and restoring epithelial integrity through apoptosis reduction [302], upregulation of antioxidant enzymes (HO‐1, NQO‐1) [279, 300, 301], and promotion of angiogenesis [290].

MSCs and their EVs additionally attenuate intestinal fibrosis (a leading cause of stricturing and surgery in CD), by downregulating TGF‐β, α‐smooth muscle actin, collagen, and fibronectin [276, 327, 328], partly through hepatocyte growth factor and TSG‐6 [329].

Finally, MSCs and MSC‐derived exosomes restore the gut microbial balance disrupted in IBD [180], enhancing diversity and abundance and shifting bacterial composition toward that of healthy mice [279, 283, 290, 325, 328, 330, 331], thereby reshaping the microbiota–metabolite–immune axis.

##### 3.2.3.2 Clinical Evidences

Clinical research on MSCs in IBD patients has mainly concentrated on CD, exploring both autologous and allogeneic MSC transplantation through local and systemic delivery methods to address complications such as refractory fistulas, strictures, and luminal disease. Autologous MSC therapies have shown promise and safety in some trials. Intravenous infusions of autologous BM‐derived MSCs (1–10 × 10^6^ cells/kg) have been demonstrated to be both feasible and well tolerated [332, 333]. Local intracolonic injection of autologous adipose‐derived MSCs has been safe and led to complete fistula healing in over half (57%) of treated patients [334, 335, 336], with only transient side effects like postprocedure pain and urinary retention [336]. Embedding autologous adipose‐derived MSCs within a bioabsorbable scaffold for fistula repair yielded encouraging results, with a high proportion of patients experiencing full clinical and radiographic healing 6 months posttreatment [337].

The majority of clinical trials have employed allogeneic MSCs, primarily from umbilical cord tissue [338] and bone marrow [339, 340, 341], with no serious adverse events reported [338, 339] for either systemic or local transplantation [340, 341].

Intravenous infusions of allogeneic MSCs (1–2 × 10^6^ cells/kg body weight weekly for 4 weeks) led to decreased DAI and Crohn's Disease Endoscopic Index of Severity scores, along with a reduction in corticosteroid requirements [338, 339]. However, one pilot open‐label study using intravenous allogeneic bone marrow MSCs in patients with severe refractory CD did not observe significant improvements in clinical or inflammatory markers (C‐reactive protein or fecal calprotectin) [342]. More targeted local therapies, such as intracolonic injections of allogeneic bone marrow MSCs (3–9 × 10^7^ cells/patient), have shown sustained reductions in fistula size over 4 years [340]. More recently, exosomes derived from UC‐MSCs were evaluated for refractory perianal fistula, with three out of five patients showing complete healing and one partial healing 6 months posttreatment, though ineffective in fibrotic tracts [343] (see Table 4).

Darvadstrocel (Cx601, Alofisel), an expanded allogeneic adipose‐derived MSC product for complex Crohn's perianal fistulas, showed benefit in the Phase III ADMIRE‐CD trial (*n*  =  212): combined remission at Week 24 was 51.5 versus 35.6% with placebo (*p*  =  0.021), with sustained benefit to 52 and 104 weeks [344, 345, 346]. However, the larger ADMIRE‐CD II (*n*  =  568) failed to meet its primary endpoint (combined remission 48.8 vs. 46.3%; *p*  =  0.571) (Takeda press release https://www.takeda.com/newsroom/statements/2024/takeda‐alofisel‐update‐2024/; [347]). No new safety signals emerged. However, following EMA review, the European Commission withdrew Alofisel's EU marketing authorization on December 13, 2024 (EMA EPAR Alofisel), and marketing in Japan ended in January 2026. The Alofisel case underscores that positive results from smaller, more homogeneous trials may not generalize to larger populations. Large, geographically diverse, biomarker‑stratified trials are now essential for durable regulatory and clinical adoption.

Studies confirm that both local and systemic infusions of MSCs have favorable safety profiles. Strong evidence supports the long‐term efficacy of local MSC injections for perianal fistulizing CD, while data on systemic MSC infusions for luminal IBD show mixed results. These inconsistencies likely stem from suboptimal standardization in MSC therapy, driven by MSC heterogeneity (due to varying tissue sources and culture conditions), and the lack of optimized protocols—particularly regarding infusion method, dose, and dosing intervals. Resolving these heterogeneity issues, rather than chasing incremental efficacy gains, is now the rate‐limiting step for clinical MSC translation in IBD; product‐standardization initiatives, potency‐assay development, and biomarker‐driven patient selection will likely matter more than the choice of cell source in determining whether the next generation of MSC trials succeeds.

### 3.3 Potential Combination Therapies

Despite substantial therapeutic advances in IBD, many patients continue to experience suboptimal treatment outcomes, underscoring a persistent limitation in therapeutic efficacy [274].

This can reflect the current absence of clear definitions for treatment optimization, treatment failure, or criteria to abandon therapy. Across trials, criteria for a response of both patients with UC and CD are heterogenous. As already underlined before, investigators use different definitions for clinical and endoscopic remission, and endoscopic response and outcomes are assessed at variable time points. Primary nonresponse to advanced therapies, such as anti‐TNF therapies, is most commonly explained by two mechanisms: pharmacokinetic problems (fast drug clearance leading to low blood levels) or pharmacodynamic failure (inflammation mediated by alternative pathways to the mechanism targeted by the allocated therapy) [348, 349]. Secondary nonresponse, or loss of response, occurs when patients initially respond to advanced therapy then subsequently lose response. For anti‐TNF agents, loss of response often results from suboptimal drug concentration, formation of antidrug antibodies [350], or a shift to new inflammation pathways [351].

Pharmacodynamic failure to anti‐TNF therapy (e.g., infliximab) has been observed in nonimmunogenic patients and with adequate trough levels who nonetheless exhibit upregulation in mucosal of IL17 and IL23 transcripts, suggesting that drug failure may be driven by alternative pathways driving inflammation, such as the IL‐23/IL‐17 axis [352].

On the other hand, suboptimal treatment outcomes can reflect the complex and multifactorial nature of IBD pathogenesis, involving dynamic interactions among the immune system (immunome), environmental exposures (exposome), host genetics (genome), and the intestinal microbiota (microbiome) [353]. Critically, these distinct failure mechanisms—immunogenic loss‐of‐response, pharmacodynamic switching to alternative cytokine axes, and microbiota‐driven inflammation refractory to immune‐pathway blockade—reveal a tripartite biological heterogeneity that single‐target monotherapy cannot accommodate. Interpreting nonresponse through this lens transforms it from a clinical inconvenience into a diagnostic signal that should guide rational, mechanism‐matched combination strategies and biomarker‐stratified trial design (developed in detail in Section 4.3).

Critical reading of the comparative‐effectiveness and biomarker literature suggests that the 30–40% rate of primary nonresponse and the 30–40% rate of secondary loss‐of‐response observed across biologic classes are not a single phenomenon, but rather the convergent end‐point of three biologically distinct failure modes. First, immunogenicity and pharmacokinetic loss‐of‐response, enriched in HLA‐DQA1*05 carriers (approximately 40% of Europeans), drive a substantial proportion of anti‐TNF secondary failures [350]. Second, a tissue cellular module consisting of IgG plasma cells, inflammatory mononuclear phagocytes and oncostatin‐M‐expressing stromal cells (the GIMATS/OSM signature), identifies a transcriptomic class of primary anti‐TNF nonresponders [106, 149]. Third, dysbiotic enterotypes characterized by Bacteroides2 expansion or by depletion of butyrate‐producing *Faecalibacterium* and *Roseburia* define a microbiota‐driven inflammatory subtype refractory to immune‐pathway blockade alone [354]. Recognizing these failure modes as distinct biological entities, rather than as a homogeneous “refractory” group, reframes the rationale for combination and cell‐based therapies. We therefore propose three biomarker‐anchored, testable hypotheses for next‐generation IBD trials. (i) Patients carrying HLA‐DQA1*05 together with high baseline tissue OSM/GIMATS are predicted to significantly benefit from early combination of anti‐TNF with an anti‐IL‐23p19 agent or with MSC‐based immunomodulation, rather than from anti‐TNF monotherapy. (ii) Patients with stricturing CD enriched for IL12B/IL23R risk alleles and an IL‐23‐dominant transcriptome should preferentially receive anti‐IL‐23p19 plus an antifibrotic strategy (HGF/TSG‐6‐rich MSC‐EVs or targeted TGF‐β‐pathway modulators), to prevent transmural progression. (iii) Patients with refractory UC harboring the *Bacteroides2* enterotype should be enrolled in combinations of gut‐selective biologics (vedolizumab, ozanimod) with microbiota‐directed interventions (FMT, defined consortia, or microbiome‐modulating MSC‐EVs). Validating these hypotheses will require adaptive, biomarker‐stratified platform trials with histologic remission (UC) and transmural healing (CD) as coprimary endpoints, shifting the field decisively from one‐size‐fits‐all combination strategies toward mechanism‐matched precision interventions.

Consequently, there is growing recognition of the need for integrated therapeutic strategies that target multiple disease axes [313, 355].

Combination therapy is already well established in IBD management. Notably, Phase III trials have demonstrated that infliximab in combination with azathioprine is superior to monotherapy for inducing corticosteroid‐free clinical remission in both CD and UC, with additional benefits including reduced antidrug antibody formation and fewer infusion reactions [356]. However, long‐term studies are still needed to fully understand its value in preventing bowel damage and disability, and its association with serious drug‐related adverse events or mortality [357].

Beyond traditional immunomodulator–biologic combinations, interest is increasing in dual biologic therapy and small molecule–biologic combinations, particularly for patients with refractory disease [358]. Preliminary evidence from small observational studies suggests that combinations such as tofacitinib with vedolizumab or ustekinumab may be effective in achieving clinical and endoscopic remission in selected individuals [359, 360]. Nonetheless, these approaches remain limited by small patient cohorts, a lack of long‐term safety data, and elevated healthcare costs [361]. Importantly, such strategies may reduce IBD‐related hospitalizations and surgical interventions, potentially improving overall patient productivity and quality of life. MSCs are emerging as a promising adjunctive therapy due to their immunomodulatory and regenerative properties [313]. Preclinical studies have explored MSCs in combination with conventional anti‐inflammatories such as sulfasalazine [362], microbiota‐directed interventions such as MIS416 [363] and VSL#3 [364], and other cell‐based therapies, such as tolerogenic DCs [365], hematopoietic stem cells [366], or Treg cells [367]. These combinations have shown additive or synergistic effects in ameliorating colitis in animal models. Notably, clinical trials of MSCs in IBD typically involve patients who continue their standard anti‐inflammatory treatments, reinforcing the feasibility of combination approaches [313]. Given the intricate pathophysiology of IBD, therapeutic strategies that incorporate MSCs alongside biologics or small molecules may better address the heterogeneity of disease mechanisms. However, well‐designed clinical trials are needed to evaluate their long‐term efficacy and safety.

On the other hand, FMT, by directly targeting intestinal dysbiosis, may represent a combination strategy to foster a healthier intestinal ecosystem, thereby supporting the sustained efficacy of biological agents and reducing the incidence of secondary nonresponse.

A key concern in the clinical application of FMT is the variability in treatment efficacy, which could compromise the reliability of outcomes [368]. While large clinical trials support a broadly acceptable safety profile for FMT, risks persist‐including the potential transfer of harmful microorganisms and microbial functions to recipients. This is especially alarming amid rising antibiotic resistance, as FMT could inadvertently transmit resistance genes and virulence factors, resulting in serious unintended consequences.

In summary, while an optimal, universal treatment for IBD remains elusive, the rationale for combination therapy is compelling, particularly in patients with inadequate monotherapy responses. A multipronged therapeutic approach, guided by molecular and cellular profiling, may represent the future of personalized IBD management.

## 4 Conclusion and Prospects

This section traces they key challenges in IBD research and management: from its multifactorial origins and limitations of preclinical models (Section 4.1), through the limitations of single‐target therapy, including incomplete remission (Section 4.2), to emerging multitarget innovations such as FMT and MSC therapies (Section 4.3), and finally to translational hurdles including standardization and regulation (Section 4.4). Across all these dimensions, the need for validated biomarkers and field‐wide consensus emerges as a prerequisite for meaningful progress.

### 4.1 Challenges in Understanding and Treating IBD

IBD exemplifies the complexity of immune‐mediated disorders, arising from a multifactorial interplay among genetic predisposition, environmental exposures, microbial imbalance, and immune dysregulation. This intricate network generates substantial clinical heterogeneity, complicating both disease modeling and therapeutic innovation. Current preclinical models, although valuable to provided unprecedented insights into disease mechanisms, fail to fully recapitulate the human condition, thereby limiting their predictive translational value and contributing to variable therapeutic outcomes.

### 4.2 Limitations of Current Therapeutic Strategies

Existing treatments predominantly target single inflammatory mediators or immune pathways. While biologics and small‐molecule inhibitors have markedly improved disease management, they frequently fail to achieve durable remission. The single‐target paradigm contributes to incomplete responses, therapeutic resistance, and heterogeneous outcomes, underscoring the need for comprehensive strategies that address the multifactorial pathogenesis of IBD.

### 4.3 Emerging Opportunities: Toward Multitarget and Personalized Approaches

Future therapeutic advances will depend on integrated, multitarget strategies capable of modulating multiple pathogenic mechanisms simultaneously. Promising interventions include the regulation of gene expression via miRNA‐124, the restoration of microbial equilibrium through FMT, and regenerative cell‐based therapies. Among these, MSC‐based therapies are particularly promising and distinct from conventional biologics. Unlike monoclonal antibodies that neutralize specific inflammatory cytokines, MSCs function as a multitarget therapeutic platform, combining immunomodulatory, anti‐inflammatory, and tissue‐reparative effects. Rational combination of such approaches with existing biologics or small molecules may provide synergistic benefits and enhance long‐term efficacy.

Patient stratification offers a complementary route to translating these mechanistic insights into clinical benefit (Table 2). Three converging biomarker themes are now mature enough to formulate testable hypotheses for the next generation of precision‐medicine trials. First, the GIMATS module and the stromal oncostatin‐M signature identify a sub‐population of anti‐TNF nonresponders enriched for plasma cells, inflammatory monocytes, and activated stromal cells [106, 149]. Patients with high baseline tissue OSM would be predicted to benefit preferentially from combinations of anti‐TNF with anti‐IL‐23 or with MSC/EV immunomodulation rather than from anti‐TNF monotherapy. Second, the HLA‐DQA1*05 allele—present in approximately 40% of Europeans—doubles the rate of immunogenicity‐driven loss of response to infliximab and adalimumab [350]; these patients are predicted to derive added benefit from concomitant immunomodulator therapy or from early non‐TNF biologic strategies. Third, microbiota enterotyping into the dysbiotic *Bacteroides2* cluster [354] and the IL12B/IL23R risk‐allele profile suggest patient subgroups in which microbiota‐modulating cotherapy (FMT, MSC‐EV‐mediated microbiome restoration) or anti‐IL‐23/antifibrotic combinations might prevent the stricturing CD phenotype.

These hypotheses are not yet supported by adequately powered prospective trials. The biomarker‐stratified PROFILE study [127] found that the prognostic T‐cell signature did not differentiate patients who benefited from early combined immunosuppression, indicating that current single‐biomarker assays are insufficient and that composite biomarker signatures integrating genetic, transcriptomic, microbial, and serological data are required. A pragmatic forward path is to design adaptive, biomarker‐stratified platform trials with transmural healing (for CD) and histologic remission (for UC)—the deep‐remission targets endorsed by STRIDE‐II) [228]—as coprimary endpoints, and to enroll cell‐based interventions (MSCs, EVs, Treg cells) alongside biologics with predefined molecular subgroup criteria. The negative ADMIRE‐CD II result reinforces this imperative: matching the therapeutic mechanism to the patient's molecular subtype, rather than testing one‐size‐fits‐all cell preparations in heterogeneous populations, is likely the decisive next step toward durable, personalized control of IBD.

### 4.4 Path Toward Clinical Translation

Realizing the potential of these emerging therapies requires rigorous translational and clinical validation. Key priorities include optimizing dosing and treatment regimens, identifying predictive biomarkers of therapeutic response, and ensuring long‐term safety. However, substantial barriers to translation persist. A primary challenge is the variability in MSC sources, particularly the functional differences between bone marrow‐derived and adipose‐derived MSCs, which can lead to divergent therapeutic outcomes. Furthermore, the lack of standardization in administration protocols (including route, frequency, and cell dosage) has led to inconsistent findings, limiting cross‐trial comparability.

Regulatory hurdles also represent a critical bottleneck. The absence of harmonized definitions for clinical response and remission, alongside the complexities of establishing consistent manufacturing and quality control standards, significantly complicates regulatory acceptance. Additional challenges include donor variability and cryopreservation techniques, both of which may influence cell potency. To fully realize the potential of MSC‐based therapies, future research must prioritize methodological standardization, long‐term follow‐up, the development of potency assays, as well as the establishment of consensus efficacy criteria to satisfy regulatory requirements.

### 4.5 Outlook

The future of IBD management lies in moving beyond inflammation control toward immune re‐education and mucosal restoration, guided by precision medicine principles. By embracing the biological complexity of IBD and leveraging advances in genomics, microbiomics, and immunology, next‐generation multitarget therapies hold the potential to achieve sustained remission and fundamentally transform patient outcomes. This paradigm shifts from single‐pathway inhibition to integrated, personalized intervention marks a decisive step forward in redefining both the scientific and clinical landscape of IBD.

## Author Contributions

Conceptualization: A.P. A.C., and O.P. Writing – original draft: A.P., A.C., F.S., L.R.L., A.R.S., and O.P. Writing – review and editing: A.P., A.C., F.S., L.R.L., A.R.S., and O.P. Funding acquisition: O.P. Supervision: A.P. and O.P. All authors have read and approved the final manuscript.

## Funding

This work was supported by Università Cattolica del Sacro Cuore (Linea D1 OP), by the Italian Ministry of Research and University (MIUR, 5 × 1000), Contributi per il funzionamento degli Enti privati che svolgono attività di ricerca—C.E.P.R. This work was supported by Ministero della Salute‐Ricerca Finalizzata Progetti Giovani Ricercatori (GR)‐Codice Progetto: GR‐2024‐12380233.

## Ethics Statement

The authors have nothing to report.

## Conflicts of Interest

The authors declare no conflicts of interest.

## Data Availability

The authors have nothing to report.
